# High plasticity of axonal pathology in Alzheimer’s disease mouse models

**DOI:** 10.1186/s40478-017-0415-y

**Published:** 2017-02-07

**Authors:** Lidia Blazquez-Llorca, Susana Valero-Freitag, Eva Ferreira Rodrigues, Ángel Merchán-Pérez, J. Rodrigo Rodríguez, Mario M. Dorostkar, Javier DeFelipe, Jochen Herms

**Affiliations:** 1 German Center for Neurodegenerative Diseases-Munich site (DZNE-M) and Center for Neuropathology and Prion Research (ZNP), Ludwig-Maximilians University, Munich, Feodor-Lynen-St 23, 81377 Munich, Germany; 2 Laboratorio Cajal de Circuitos Corticales, Centro de Tecnología Biomédica, Universidad Politécnica de Madrid, Madrid, Spain; 3 Departamento de Arquitectura y Tecnología de Sistemas Informáticos, Escuela Técnica Superior de Ingenieros Informáticos, Universidad Politécnica de Madrid, Madrid, Spain; 4 Instituto Cajal, Consejo Superior de Investigaciones Científicas, Madrid, Spain; 5 Centro de Investigación Biomédica en Red sobre Enfermedades Neurodegenerativas (CIBERNED), ISCIII, Madrid, Spain; 6 Munich Cluster of Systems Neurology (SyNergy), Ludwig-Maximilians University, Munich, Germany; 7 Departmento de Psicobiología, Facultad de Psicología, Universidad Nacional de Educación a Distancia (UNED), C/Juan del Rosal 10, 28040 Madrid, Spain

**Keywords:** Alzheimer’s disease, Dystrophic neurites, FIB/SEM microscopy, Three-dimensional, Two-photon microscopy

## Abstract

Axonal dystrophies (AxDs) are swollen and tortuous neuronal processes that are associated with extracellular depositions of amyloid β (Aβ) and have been observed to contribute to synaptic alterations occurring in Alzheimer’s disease. Understanding the temporal course of this axonal pathology is of high relevance to comprehend the progression of the disease over time. We performed a long-term in vivo study (up to 210 days of two-photon imaging) with two transgenic mouse models (dE9xGFP-M and APP-PS1xGFP-M). Interestingly, AxDs were formed only in a quarter of GFP-expressing axons near Aβ-plaques, which indicates a selective vulnerability. AxDs, especially those reaching larger sizes, had long lifetimes and appeared as highly plastic structures with large variations in size and shape and axonal sprouting over time. In the case of the APP-PS1 mouse only, the formation of new long axonal segments in dystrophic axons (re-growth phenomenon) was observed. Moreover, new AxDs could appear at the same point of the axon where a previous AxD had been located before disappearance (re-formation phenomenon). In addition, we observed that most AxDs were formed and developed during the imaging period, and numerous AxDs had already disappeared by the end of this time. This work is the first in vivo study analyzing quantitatively the high plasticity of the axonal pathology around Aβ plaques. We hypothesized that a therapeutically early prevention of Aβ plaque formation or their growth might halt disease progression and promote functional axon regeneration and the recovery of neural circuits.

**Electronic supplementary material:**

The online version of this article (doi:10.1186/s40478-017-0415-y) contains supplementary material, which is available to authorized users.

## Introduction

Alzheimer’s disease (AD) is typically associated with a set of neuronal cytoskeletal alterations – the formation of neurofibrillary tangles (NFTs), neuropil threads and dystrophic neurites, which are associated with dendritic spine and synapse loss, as well as neuronal degeneration (e.g., [2, 42, 53, 61]). These pathological changes develop in a characteristic spatiotemporal progression across the cerebral cortex and other brain regions in AD patients [12] and AD mouse models [10]. Dystrophic neurites are swollen and tortuous neurites, which were originally detected by Alois Alzheimer because of their argyrophilia [1]. They have a variable morphology and composition depending on the pathological stage of AD [44, 51, 58, 60, 62]. They are closely associated with extracellular deposits of amyloid β (Aβ), known as “Aβ plaques”, which represent another hallmark of AD pathology. Dystrophic neurites are normally formed in axons [18, 24, 25, 36, 38, 57, 58, 62]. From now on we will refer to axonal dystrophies as AxDs.

Synaptic loss is the major neurobiological basis of cognitive dysfunction in AD. Synaptic failure is an early event in the pathogenesis that is already clearly detectable in patients with mild cognitive impairment (MCI), a prodromal state of AD. Compelling evidence suggests that different forms of Aβ peptide and abnormal phosphorylated tau induce synaptic loss in AD and transgenic mice models [6]. Synaptic breakdown in AD mouse models with no relation to amyloid plaques but as a consequence of high level of soluble amyloid beta has been reported [3, 4]. Aβ plaques are associated to alterations of dendrites and axons that are in contact or in the proximity to them, and with a clear decrease of synapses. The majority of studies has been focused on alterations of dendrites in contact with Aβ plaques [8, 9, 28, 32–35, 43, 52, 53, 59]. However, less attention has been paid to the alterations of axons [2]. This is unfortunate since the loss of synapses found in or around Aβ plaques could be related to alterations of postsynaptic targets (dendrites), presynaptic elements (axons) or both.

For this reason, we consider that understanding the temporal course of the axonal pathology is of high relevance to comprehend the progression of the disease over time and define possible therapeutic targets and the window time where a treatment might be effective. The dystrophic pathology is one of the alterations of the disease that has been better resembled in the animal models [2, 13, 53, 59]. Previous in vivo studies have been undertaken to analyze the dystrophic pathology over time [13, 15, 23, 53, 59]. One of the main findings from these studies was that the elimination rates were significantly higher than the formation rates, suggesting that there is a gradual net loss of neuronal structures over time near Aβ plaques, causing a permanent disruption of neuronal connections [59]. Furthermore, it has been reported that the dystrophic pathology is reversible with an anti-Aβ antibody treatment [13] and curcumin [23]. However, these studies did not perform either a detailed quantitative analysis of the observed changes or a long-term study of the dystrophic pathology (the longest was only 35 days). Moreover, the relationship of specific axonal dystrophic changes to Aβ accumulation has not been addressed. In the present work, we performed a detailed long-term study (of up to 210 days and weekly imaging) focusing on the formation, development and elimination of AxDs with the aim of examining the plasticity of AxDs and their potential to be reversed. To achieve these objectives, we have used two-photon in vivo imaging and electron microscopy including transmission electron microscopy (TEM) and focused ion beam/scanning electron microscopy (FIB/SEM).

## Materials and methods

### Animals and housing

Mouse lines Amyloid Precursor Protein – Preseniline 1 (APP-PS1) (dE9) [30], APP-PS1 [48] and the Green Fluorescent Protein-M (GFP-M) [19] were used in this study. The dE9 and GFP-M lines were purchased from The Jackson Laboratory (Bar Harbor, USA). The APP-PS1 mice were provided by Matthias Jucker (University of Tübingen and German Center for Neurodegenerative Diseases, Tübingen, Germany). Heterozygous dE9 and APP-PS1 mice were crossed with heterozygous GFP-M mice resulting in triple transgenic dE9xGFP-M and APP-PS1xGFP-M mice, which were inbred. Heterozygous triple transgenic mice of mixed gender were used for experiments at the ages indicated below. Mice were group-housed under pathogen-free conditions until surgery, after which they were singly housed in standard cages with food and water ad libitum. The studies were carried out in accordance with an animal protocol approved by the Ludwig-Maximilians-University Munich and the government of Upper Bavaria (Az. 55.2-1-54-2531-188-09).

### Two-photon in vivo imaging

For in vivo imaging, a chronic cranial window was prepared as described previously [22]. Surgery was performed in six 6-month-old dE9xGFP-M and seven 2-month-old APP-PS1xGFP-M mice. In vivo imaging began after a 4–5-week post-surgery recovery period, using an LSM 7 MP setup (Zeiss) equipped with a MaiTai laser (Spectra Physics). Around 24 h before imaging, Methoxy-X04 (0.4 to 2.4 mg/Kg body weight, Xcessbio, San Diego, CA, USA) was intraperitoneally injected to visualize Aβ plaques in vivo [31]. Imaging was performed once a week for 24 weeks in dE9xGFP-M mice and for 30 weeks in the APP-PS1xGFP-M mice. In the dE9 model, the imaging began when the mice were around 7 months-old (the age corresponding to the initial stage of the amyloid pathology) and was prolonged until they were approximately 13 months-old (corresponding to the advanced stage of the disease). In the APP-PS1 mouse, the imaging began when the mice were around 3 months-old (the age corresponding to the initial stage of the amyloid pathology) and was prolonged until they were approximately 10 months-old (which corresponds to the advanced stage of the disease).

Two-photon excitation of Methoxy-X04-labeled Aβ plaques was performed at 750 nm and the signal was detected using a short pass (SP) 485 nm filter. Two-photon excitation of GFP-expressing neuronal structures was performed at 880 nm and the signal was detected using a bandpass (BP) 500–550 nm filter. To exclude false positive fluorescent spots from the analysis, we also recorded emissions at 590–650 nm. These auto-fluorescent spots were found both in the neuropil and within neuronal and glial cells (Additional file 1). A × 20 1.0 NA water-immersion objective (Zeiss) was used. Stereological coordinates were used to locate the somatosensory cortex [29]. Overview images were taken at low resolution (logical size 512 × 512 pixels; physical size x, y, z: 424.3 × 424.3 × 300 μm; z-step = 3 μm) to a depth of 300 μm (supragranular layers) to find the same position over time. At least 2–3 overviews were taken per animal at each imaging session. Note that performing long-term in vivo two-photon imaging weekly during near 6 months is challenging. Although more imaging positions were acquired only those that were successfully imaged during the whole time period were used for the quantitative analysis. These numbers are shown in Additional file 2. Two types of images were taken to perform the analysis:

i) The three-dimensional (3D) reconstruction of AxDs over time: High magnification images (logical size 512 × 512 pixels; physical size x, y, z: 84.9 × 84.9 × 40–60 μm; z-step = 1 μm) of single Aβ plaques stained with Methoxy-X04 and the GFP-expressing neurites around them (46 GFP-expressing axons around 6 Aβ plaques in the dE9xGFP-M mouse model (*n* = 6), 10 of which became dystrophic and were 3D reconstructed; 58 GFP-expressing axons around 6 Aβ plaques were followed in the APP-PS1xGFP-M mouse model (*n* = 7), 16 of which became dystrophic and were 3D reconstructed). Care was taken to ensure similar fluorescence levels in space and time.
ii) The spatiotemporal relationship between Aβ plaques and AxDs: Panoramic high resolution images (logical size 1400 × 1400 pixels; physical size x, y, z: 202.3 × 202.3 × 39.9–50.1 μm; z-step = 0.3 μm) showing several Aβ plaques stained with Methoxy-X04 and GFP-expressing neurites near and far from them (33 Aβ plaques and 52 AxDs were followed over time in the APP-PS1xGFP-M mouse model (*n* = 7)). AxDs were not 3D reconstructed at all time points, but rather only on those days when the volume of the AxD was visually observed to be largest. Thus, we recorded “the maximum volume data” over time. Moreover, the day of appearance and disappearance and the type of axon in which the AxD appeared was also annotated for every single AxD. Care was taken to ensure similar fluorescence levels in space and time.

### Electron microscopy preparation and TEM and FIB/SEM imaging

A correlative two-photon in vivo imaging and TEM or FIB/SEM microscopy method was used to analyze the ultrastructure of the same Aβ plaques (and AxDs around them) as those previously studied in vivo [11]. Briefly, after the final in vivo imaging session, three dE9xGFP-M mice were transcardially perfused with 2% paraformaldehyde and 2.5% glutaraldehyde in 0.12 M PB, pH 7.4. Later, regions of interest in a thick section cut from the window region were marked by laser, using the two-photon-laser system according to the Near Infrared Branding (NIRB) technique [7]. This thick section was resectioned in thinner sections of 50 μm with a Leica Vibratome (VT1200, Leica Microsystems, Wetzlar, Germany). After the cutting, sections were analyzed again under the two-photon microscope to find those slices where the marked regions of interest were present. Selected 50 μm sections containing the regions of interest were postfixed in 2.5% glutaraldehyde/2% paraformaldehyde in 0.1 M cacodylate buffer for 1 h, treated with 1% osmium tetroxide in 0.1M cacodylate buffer for 1 h, dehydrated, and flat embedded in Araldite resin [17, 41]. The postfixation, dehydration and embedding steps were done with a laboratory microwave oven with a vacuum chamber and cooling stage (Ted Pella, Redding, CA, USA).

In those samples that were analyzed by TEM, plastic-embedded sections were studied by correlative light and electron microscopy, as described in detail elsewhere [17]. Briefly, sections were photographed under the light microscope and then serially cut into semithin (2-μm thick) sections on a Leica ultramicrotome (EM UC6, Leica Microsystems). The semithin sections were stained with 1% toluidine blue in 1% borax, examined under the light microscope, and then photographed to locate the NIRB-marked region of interest. Serial ultrathin sections (50- to 70-nm thick) were obtained from selected semithin sections on a Leica ultramicrotome, and collected on formvar-coated single-slot nickel grids and stained with uranyl acetate and lead citrate. Digital images were captured at different magnifications on a Jeol JEM-1011 TEM (JEOL Inc., MA, USA) equipped with an 11 Megapixel Gatan Orius CCD digital camera.

In those samples that were analyzed by FIB/SEM, semithin sections (1-μm thick) were obtained by means of a Leica ultramicrotome from the surface of the block until the most superficial NIRB marks around the region of interest were reached (Additional file 3). The blocks containing the embedded tissue were then glued onto aluminum sample stubs using conductive carbon adhesive tabs (Electron Microscopy Sciences, Hatfield, PA). All surfaces of the Araldite blocks, except for the top surface containing the sample, were covered with colloidal silver paint (Electron Microscopy Sciences, Hatfield, PA) to prevent charging artifacts. The stubs with the mounted blocks were then placed into a sputter coater (Emitech K575X, Quorum Emitech, Ashford, Kent, UK) and were coated with platinum for 10 s to facilitate charge dissipation. The marks were still visible on the surface of the block with the FIB/SEM. The ultrastructural 3D study of these samples was carried out using a combined FIB/SEM microscope (Neon40 EsB, Carl Zeiss NTS GmbH, Oberkochen, Germany). The sequential automated use of alternating FIB milling and SEM imaging allowed us to obtain long series of images representing 3D sample volumes of selected regions. Images of 2048 × 1536 pixels at a resolution of 6.203 nm per pixel were taken; each individual photomicrograph therefore covered a field of view of 12.7 × 9.5 μm. The layer of material milled by the FIB in each cycle (equivalent to section thickness) was 30 nm. A total of 305 serial sections were obtained. Thus, the physical size of the stack was (x, y, z) 12.7 × 9.5 × 9.15 μm.

### Images, data processing and statistics

The deconvoluted two-photon images (AutoQuantX2, Media Cybernetics) were processed later by means of Imaris software (Bitplane AG, Zurich, Switzerland) to obtain the 3D reconstructions of the dystrophic axons and the Aβ plaques, as well as the volumes of each of them at the different time points. For the alignment (registration) of the stack of FIB/SEM images, we used Fiji (http://fiji.sc). Reconstruct Software v1.1.0.0 [21] was used to carry out the 3D reconstruction of the AxDs and the microglial cell.

Regarding Aβ plaques: the images were analyzed as time series of 3D images in Imaris. First, images were contrast-normalized (i.e., based on the average and standard deviation of the 3D stack intensities). Plaque volumes were extracted by 3D-surface-rendering with background subtraction and a threshold of 500. Newly formed Aβ plaques were tracked back to the first time point when they appeared and were only assessed when present for at least 3 time points. Regarding AxDs: they were manually segmented in the images stacks. Only those AxDs and parent axons that were present in the whole imaging stack at all time points were reconstructed. An axonal segment was considered dystrophic when its volume was double that of the non-dystrophic axonal segment. When possible, non-dystrophic axonal volume was calculated as the average of three measurements at three different time points for the same axonal segment that would later go on to show the AxD. When the AxD was already present from the first day of observation, non-dystrophic segments of the same axon outside the Aβ plaque were averaged at three different time points. In all cases, reference non-dystrophic axonal segments had the same length as the maximal segment affected by the AxD (Additional file 4).

Photoshop CS6 (Adobe Systems Inc., San José, CA, USA) software was used to generate the figures.

All data sets were tested for normality with the Kolmogorov-Smirnov and D’Agostino and Pearson omnibus normality tests with a significance level set to *p* = 0.05, before the appropriate parametric or non-parametric statistical comparison test was carried out with GraphPad Prism 5.04 (GraphPad Inc., La Jolla, CA, USA).

## Results

### Kinetics of formation, development and elimination of AxDs: 3D reconstructions

**Fig. 1:**
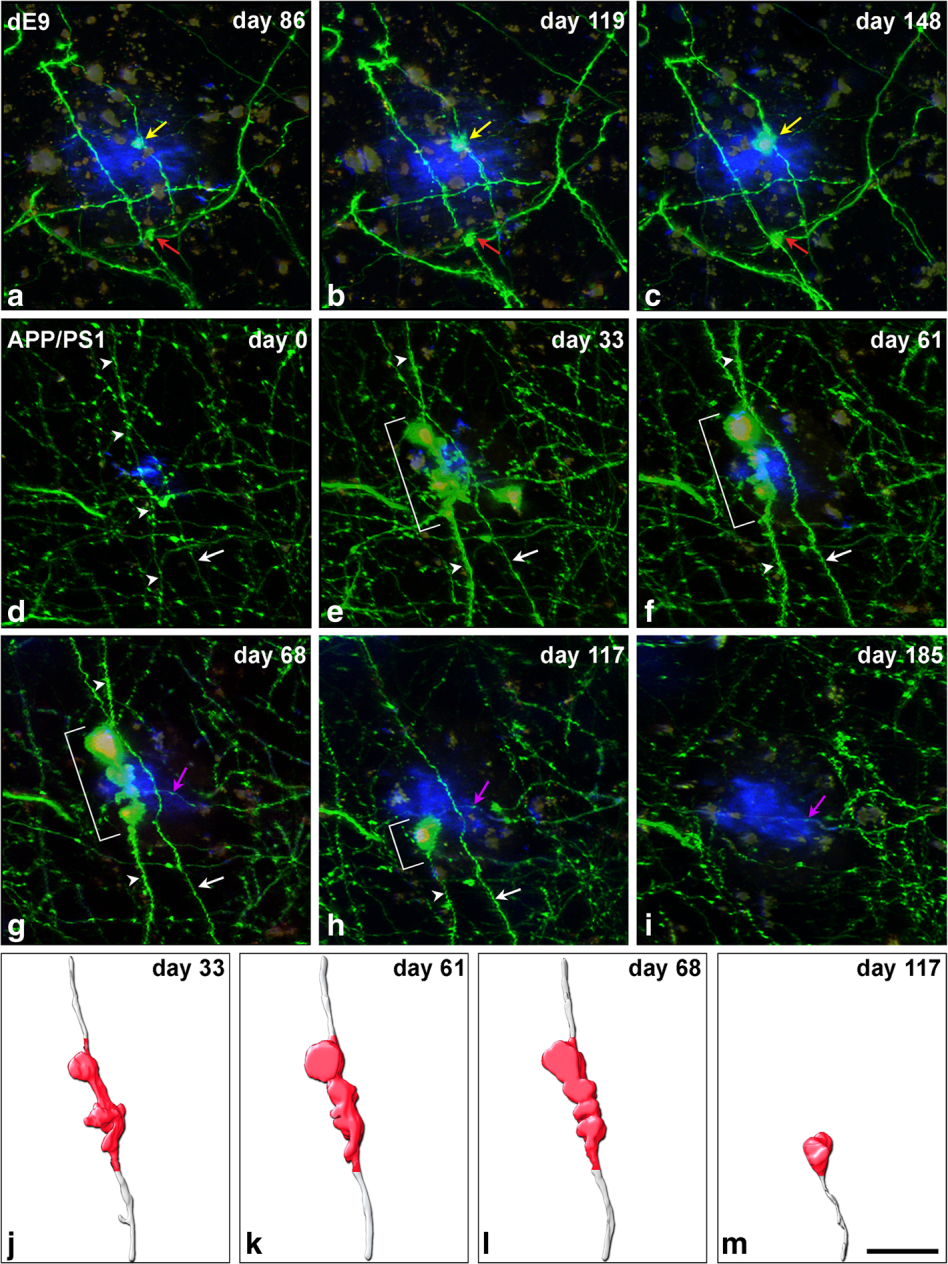
Intra- and inter-size variations of AxDs over time. (**a**-**c**), Maximum projection (40 optical sections, z-step = 1 μm) of a stack of images taken with a two-photon microscope in the somatosensory cortex of a dE9 mouse at three different time points. Small AxDs (*yellow* and *red arrows*, dys 6 and 7, respectively) in GFP-expressing axons (*green*) are present around an Aβ plaque stained with Methoxy-X04 (*blue*). (**d**-**i**), Maximum projection (32 optical sections, z-step = 1 μm) of a stack of images taken with the two-photon microscope in the somatosensory cortex of the APP-PS1 mouse at six different time points. A large AxD (*brackets* in **e**-**h**; dys 4) in a GFP-expressing axon (*green*; *arrowheads*) is present around an Aβ plaque stained with Methoxy-X04 (*blue*). This plaque was observed growing in size from its birth (**d**) to maturation (**i**). There is a degeneration of the distal part of the axon and the AxD remains at the edge of the proximal part of the axon (**h**). On the final day of imaging, the whole axon had disappeared (**i**). AxDs in panels **a**-**c** do not show strong variations in size and shape over time as compared to the AxD in panels **d**-**i**, and numerous axons and dendritic processes do not become dystrophic. There is an axon segment that does not become dystrophic and disappears (*white arrow*). (**j**-**m**), 3D reconstructions of the AxD showed in images **e**-**h**, respectively, using Imaris software. The dystrophic segment of the axon is shown in *red* and this is the portion of the axon that was used to calculate the AxD volume (Fig. 3b). The days shown refer to the number of days after day 0 (when imaging began). *Purple arrows* in **g**-**i** point out a re-growing axon that is also shown in greater detail in Fig. 5. *Scale bar* (in **m**): 19.5 μm in **a**-**m**

**Fig. 2:**
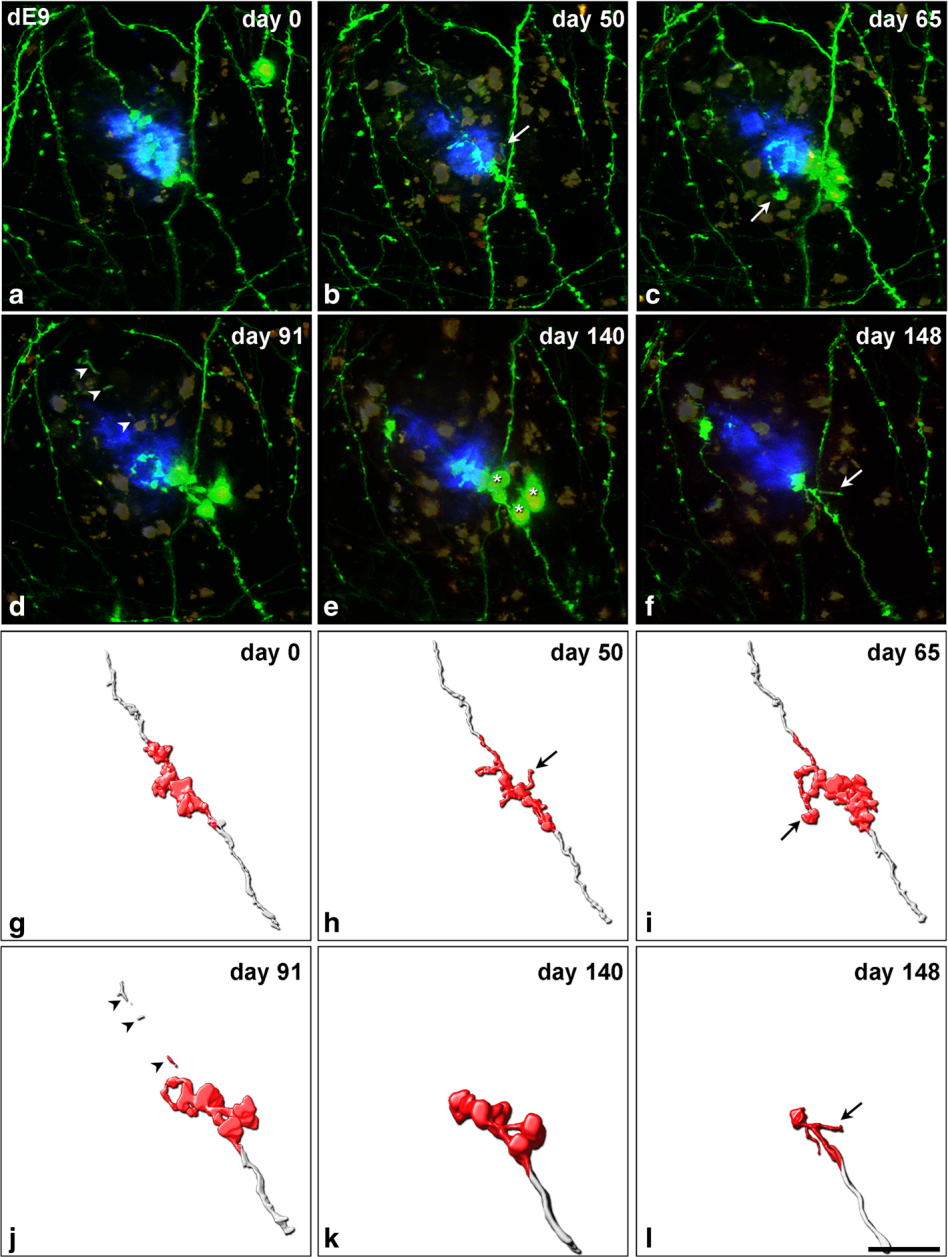
Three-dimensional reconstructions of AxDs. (**a**-**f**), Images obtained (over 148 days) of the same dystrophic axon expressing GFP (dys 1) in contact with an Aβ plaque stained with Methoxy-X04 (*blue*) in the supragranular layers of the somatosensory cortex of a dE9 mouse (two-photon microscopy). (**g**-**l**), Three-dimensional reconstructions of the AxD showed in panels **a**-**f**, respectively, using Imaris software. On day 91 (**j**) the degeneration of the distal part of the axon (*arrowheads*) begins. This degenerative process is completed in the successive days but the AxD remains at the end of the cut axon (**k**, **l**). Note that the AxD presents numerous changes in volume and shape. The existence of more than one swollen varicosity (*asterisks* in **e** show an example) and short axonal sprouting can be identified (*arrows* in **b**, **c**, **f**, **h**, **i** and **l**). Note that the dystrophic segment of the axon is shown in *red* and from this part the numerical AxD volume was calculated and plotted in Fig. 3a. The days shown refer to the number of days after day 0 (when imaging began). *Scale bar* (in **l**): 19.5 μm in **a**-**l**

**Fig. 3:**
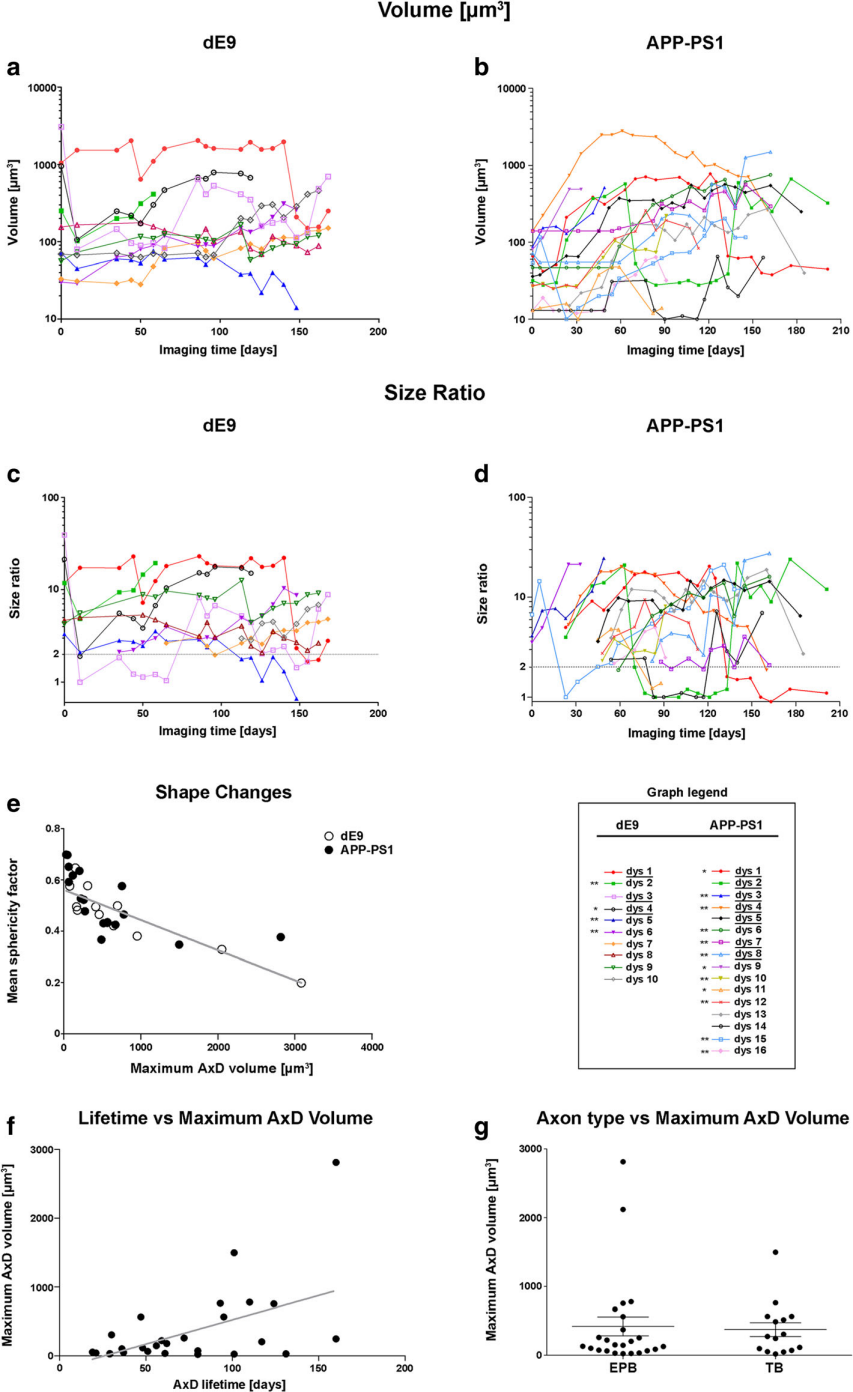
Volume and morphological changes of in vivo AxDs over time. (**a**, **b**), Graphs showing the changes in volume of the different AxDs studied over time in the dE9 (**a**) and the APP-PS1 (**b**) mice. (**c**, **d**), Size ratio indicates the ratio between the volume of an AxD and the volume of its equivalent non-dystrophic axonal segment. Graphs correspond to the same AxDs represented in **a**, **b**, respectively. With the aim of simplifying the graph visualization, the AxD size ratio was plotted only from the imaging day in which the AxD became dystrophic (size ratio ≥2, *dashed line*). Note that in **a**-**d** the scale has been transformed to Log 10 to illustrate that volume values of larger and smaller AxDs can be very similar at some time points. The days shown refer to the number of days after day 0 (when imaging began). Graph legend: *Asterisks* refer to those AxDs that disappear at the end of the imaging period (*one asterisk* means the parental axon stays and *two asterisks* mean that the AxD disappears due to the loss of the parental axon); *underlined* AxDs (dys) are those that show morphology changes (more than one swollen varicosity of irregular shape and new short axonal segments). (**e**), Correlation between the mean sphericity value over time and the maximum volume that the AxD reaches. Larger AxDs tend to be more complex, non-spherical shapes (Pearson’s r: −0.7366, *p* < 0.0001). (**f**), Correlation between the AxD lifetime and the maximum AxD volume in the APP-PS1 mouse. Larger AxDs tend to have longer lifetimes (Pearson’s r: 0.4974, *p* = 0.0071). (**g**), Comparison between the axon type (*EPB* en passant bouton axons, *TB* terminal bouton axons) and the maximum volume that the AxD reaches in the APP-PS1 mouse. The size of AxDs is not related to the type of axon in which they are formed (Mann–Whitney U: 163.0; *p* = 0.6339)

In the dE9 mouse, we observed a total of 46 axonal segments located not further than 40 μm from the border of the adjacent Methoxy-X04-stained amyloid plaques. Out of all of these axonal segments, we detected the formation of AxDs in only 22% of them (*n* = 10 AxDs; all were reconstructed with Imaris software) (Figs. 1, 2 and 3). In the APP-PS1 mouse, we examined a total of 58 axonal segments located not further than 40 μm from the border of the adjacent Methoxy-X04-stained amyloid plaques. Out of all these axonal segments, we only detected the formation of AxDs in 28% of them (*n* = 16 AxDs, all were reconstructed with Imaris software) (Figs. 1 and 3). We observed that a given AxD presented size variations over time (intra-size variations) and distinct AxDs could have very different sizes (inter-size variations) (Figs. 1 and 2). Due to this heterogeneity, we performed a detailed quantitative study of the morphological changes that take place and the kinetics of formation, development and elimination of single AxDs over time. Each AxD was independently named and they are referred to in the text and graphs as “dys 1–10” in the dE9 mouse and “dys 1–16” in the APP-PS1 mouse. Axons of control mice (GFP-M) displayed unchanged morphology after long-term in vivo two-photon imaging (observations are not shown).

Using Imaris software, 3D reconstructions of the AxDs were performed and it was possible to quantify their volume and study their morphological changes over time (Figs. 1 and 2).

#### Morphological changes of AxDs: size and shape

**Fig. 4:**
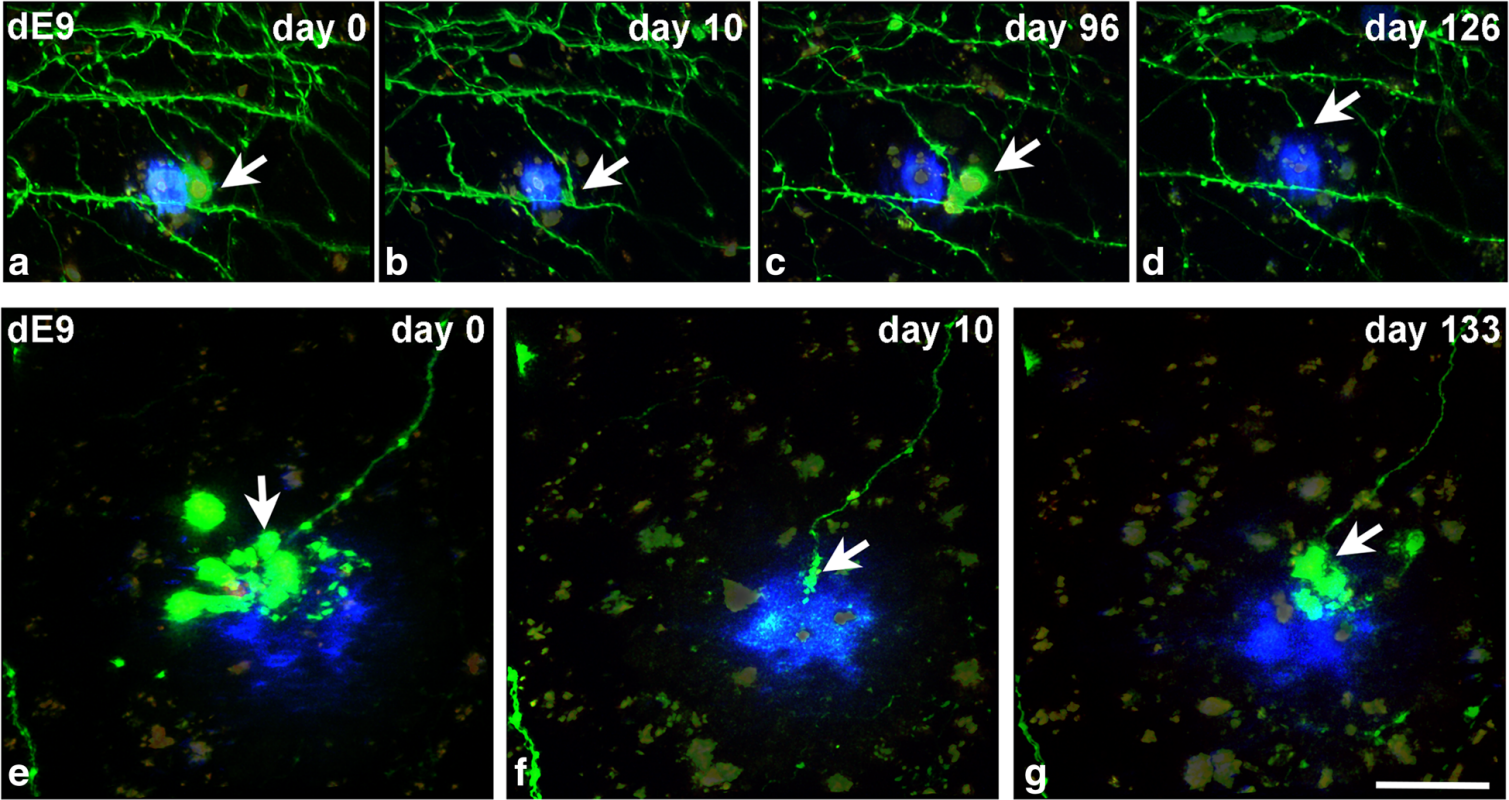
Re-formation of AxDs. Dendrites and axons expressing GFP (*green*) in contact with Aβ plaques stained with Methoxy-XO4 (*blue*) in the supragranular layers of the somatosensory cortex of the dE9 mouse (two-photon microscopy). (**a**-**d**), Maximum projection of images taken around an Aβ plaque (40 images, z = 1 μm) at different time points. The AxD (dys 4; *arrow*) is smaller on day 10 (**b**), and has disappeared on day 126 (**d**). Notice that the parental axon on day 126 is shortened. (**e**-**g**), Maximum projection of images taken around another Aβ plaque (40 images, z = 1 μm) at different time points. The AxD (dys 3; *arrow*) disappeared on day 10 (**f**) but the parental axon remains. A new large AxD is generated at the same point on day 133. The days shown refer to the number of days after day 0 (when imaging began). *Scale bar* (in **g**): 25.9 μm in **a**-**d** and 19.5 in **e**-**g**

AxDs were highly variable in terms of their size both in the dE9 and the APP-PS1 models. AxDs sizes varied between 45 and 3081 μm^3^ in the dE9 model and between 25 and 2814 μm^3^ in the APP-PS1 model (Fig. 3). Moreover, AxDs did not grow continuously; indeed their volume grew and decreased over time. Changes in volume were more prominent in the larger AxDs than in the smaller ones. For example, dys 3 in the dE9 model ranged between 159 and 3081 μm^3^ (Figs. 3a and 4e–g), while smaller AxDs showed less pronounced changes (e.g., dys 5 in the dE9 model only changed between 45 and 76 μm^3^; Fig. 3a). Thus, it can be observed that larger AxDs at some point are similar in size to those AxDs that are smaller over their whole lifetime.

When we calculated the ratio between the volume of an AxD and the volume of the non-dystrophic axonal segment of the same AxD (size ratio) (Fig. 3c, d; Additional file 4), we observed that the volume increase of the AxDs ranged between 2 and 39 times in the dE9 model and between 2 and 35 times in the APP-PS1 model (Table 1).

**Table 1:** Characteristics of the 3D reconstructed AxDs

	dE9xGFP-M								
	Type of axon	Lifetime (days)	Disappearance of AxDs at the end of the imaging period	Disappearance of the parent axon	Reformation of AxDs	Maximum AxD volume (μm^3^)	Maximum AxD size ratio	Axonal sprouting	
Dys 1	TB	>168	*No*	*No*	*No*	2049	22.98	Yes	
Dys 2	EPB	>75	Yes	Yes	*No*	414	19.40	*No*	
Dys 3	EPB	3a: >10 3b: 62 3c: >6	*No*	*No*	Yes	3a: 3081 3b: 646 3c: 697	3a: 39 3b: 8.18 3c: 8.82	Yes	
Dys 4	TB	4a: >10 4b: 116	Yes	*No*	Yes	4a: 952 4b: 789	4a: 21.3 4b: 17.64	Yes	
Dys 5	EPB	>155	Yes	Yes	*No*	76	3.57	*No*	
Dys 6	EPB	120	Yes	Yes	*No*	310	10.33	*No*	
Dys 7	EPB	>110	*No*	*No*	*No*	150	4.83	*No*	
Dys 8	EPB	>168	*No*	*No*	*No*	176	5.33	*No*	
Dys 9	EPB	>168	*No*	*No*	*No*	165	12.58	*No*	
Dys 10	EPB	>49	*No*	*No*	*No*	458	6.83	*No*	
	APP-PS1xGFP-M								
	Type of axon	Lifetime (days)	Disappearance of AxDs at the end of the imaging period	Disappearance of the parent axon	Reformation of AxDs	Maximum AxD volume (μm^3^)	Maximum AxD size ratio	Axonal sprouting	Regrowth
Dys 1	EPB	110	Yes	*No*	*No*	778	20.2	Yes	*No*
Dys 2	EPB	2a: 47 2b: >88	*No*	*No*	Yes	2a: 565 2b: 669	2a: 9.81 2b: 35.20	Yes	*No*
Dys 3	TB	>63	Yes	Yes	*No*	514	24.49	Yes	*No*
Dys 4	EPB	160	Yes	Yes	*No*	2814	20.14	Yes	*No*
Dys 5	TB	>138	*No*	*No*	*No*	565	14.77	Yes	*No*
Dys 6	EPB	124	Yes	Yes	*No*	755	16.01	*No*	*No*
Dys 7	TB	95	Yes	Yes	*No*	565	4.03	Yes	*No*
Dys 8	TB	101	Yes	Yes	*No*	1496	27.46	Yes	*No*
Dys 9	TB	>47	Yes	*No*	*No*	512	21.30	*No*	Yes
Dys 10	EPB	59	Yes	Yes	*No*	221	8.10	*No*	*No*
Dys 11	EPB	51	Yes	*No*	*No*	64	5.00	*No*	*No*
Dys 12	EPB	72	Yes	Yes	*No*	259	9.45	*No*	*No*
Dys 13	TB	>131	*No*	*No*	*No*	274	18.84	*No*	Yes
Dys 14	EPB	14a: 29 14b: >40	*No*	*No*	Yes	14a: 32 14b: 65	14a: 2.45 14b: 7.20	*No*	*No*
Dys 15	EPB	15a: >23 15b: 117	Yes	Yes	Yes	15a: 116 15b: 203	15a: 14.43 15b: 21.05	*No*	*No*
Dys 16	TB	37	Yes	Yes	*No*	48	4.80	*No*	*No*

In some cases, both in the dE9 and the APP-PS1 mice, we observed significant changes in the shape of the AxDs and the formation of more than one swollen varicosity of irregular shape with new short axonal segments leaving from the dystrophic structures (axonal sprouting) (Fig. 2). This phenomenon was observed in those AxDs reaching larger sizes (greater than 500 μm^3^) —*n* = 3 in the dE9 and *n* = 7 in the APP-PS1 mice, see Table 1 and Fig. 3— but was not seen in the smaller ones that normally remained as single spherical swollen varicosities (Fig. 1a-c). To quantify this observation, we estimated the mean sphericity factor of each AxD over time. The sphericity factor, defined as the ratio of the surface area of a sphere to the surface area of the structure analyzed (both with the same volume) provides a quantitative record of the morphological complexity of the 3D-reconstructed AxDs, since spherical objects would yield a sphericity value close to 1, while more complex shapes with larger surface-to-volume ratios would yield progressively lower values. We found an inverse correlation (Pearson’s *r*: −0.7366, *p* < 0.0001; Fig. 3e) between the sphericity and the maximum volume that the AxD reaches, so larger AxDs showed smaller sphericity factors and vice versa. Thus, larger AxDs tend to be complex, non-spherical shapes.

#### Axonal sprouting: re-growth phenomenon

**Fig. 5:**
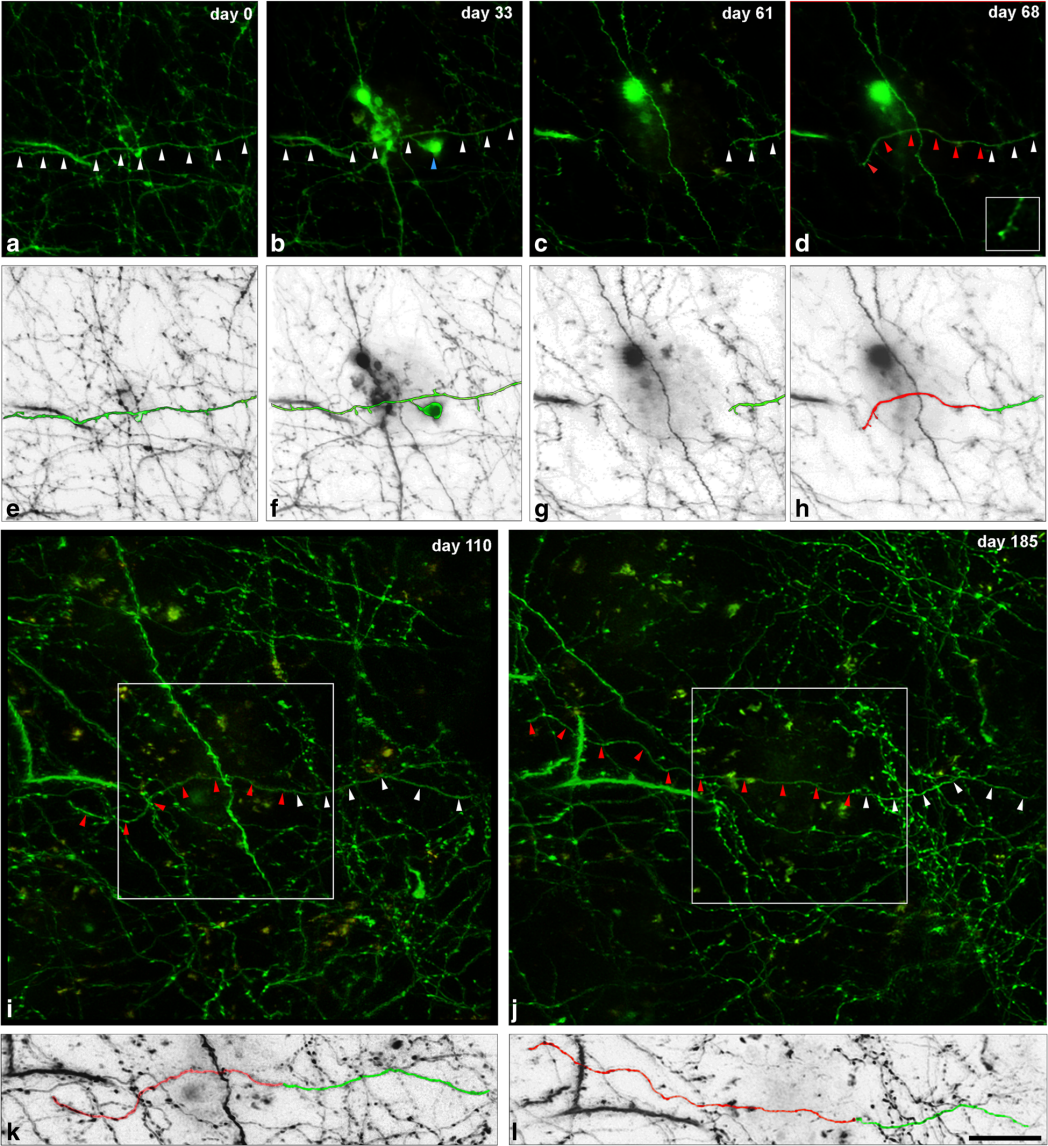
Re-growing phenomenon in a dystrophic axon. (**a**-**d**), Maximum projection of a stack of images taken in the supragranular layers of the somatosensory cortex of the APP-PS1 mouse at four different time points (two-photon microscopy). To facilitate the visualization of the axon of interest, only those optical sections where this axon was present were used for the maximum projections (32 sections in **a**, 30 in **b**, 10 in **c** and 15 in **d**; z-step: 1 μm). Panels **a**-**d** correspond to the same regions and days as those also illustrated in Fig. 1d-g. In day 61 (**c**), the distal part of the axon (*white arrowheads*) was lost just before the dystrophic part (dys 9, *blue arrowhead* in **b**). In day 68 (**d**), the axon starts to re-grow (*red arrowheads*). The *inset* in **d** shows the growth cone. (**e**-**h**), Schematic representation from images **a**-**d**, respectively, showing the axon of interest (*green*) and the re-growth segment (*red*). (**i**, **j**), Maximum projection of a stack of lower magnification images (89 sections in **i** and 98 in **j**; z-step: 0.7 μm), showing that the new axon segment (in **d**) can re-grow (*red arrowheads*) longer distances over time (re-growth segment: 73.9 μm in **i** and 104.5 μm in **j**). The *square* delimits the size of the regions shown in **a**-**d**. (**k**, **l**), Schematic representation from images **i**-**j**, respectively, showing the axon of interest (*green*) and the re-growth segment (*red*). Note that the re-growth axonal segment has changed its trajectory whereas the original axon segment maintains the original trajectory. The days shown refer to the number of days after day 0 (when imaging began). *Scale bar* (in **l**): 24 μm in **a**-**h**, 11.6 μm in **d** (*inset*) and 20.6 μm in **i**-**l**

In the case of the APP-PS1 mouse only, the formation of new long axonal segments in dystrophic axons (re-growth phenomenon, *n* = 3 (out of 17)) was observed (Fig. 5 and Additional file 5). These new axonal segments were observed either (i) leaving from a dystrophic structure (*n* = 1 (dys 13)) with a maximum observed length of the new axonal segment of 53 μm or (ii) re-growing from axons that were previously sectioned at a dystrophic point (*n* = 2) with a maximum observed length of the new axonal segments of 104.5 (dys 9, Fig. 5) and 32 μm (this AxD was not 3D reconstructed, but is shown in Additional file 5). The re-grown segment followed a different trajectory from the previously existing axon segment (Fig. 5 and Additional file 5, Table 1).

#### Lifetime and elimination of AxDs

We found that on average AxDs had a very long lifetime. It was common to find AxDs that were present for more than 100 days both in the dE9 and the APP-PS1 mice —*n* = 7 (out of 10) and *n* = 7 (out of 16), respectively— (see Table 1). In the APP-PS1 mouse, it was feasible to analyze a larger number of AxDs (see next section and Additional file 6). In this case, the average lifetime of AxDs was 76.43 ± 7.8 days (*n* = 28) —not taking into account those AxDs that were present on the first and/or last day of imaging. Moreover, we found a correlation (Pearson’s *r*: 0.4974, *p* = 0.0071) between the AxD lifetime and the maximum volume that the AxD reaches, that is, larger AxDs had longer lifetimes and vice versa (Fig. 3f).

When AxD loss occurred, it happened in two ways:

1. Loss of the whole axon where the AxD was present (Figs. 1d–i and 3, Table 1). In addition, around Aβ plaques, both normal-looking axons and dystrophic axons could disappear (see Fig. 1d–i). However, we cannot rule out the possibility of the normal axon being dystrophic at a segment close to an Aβ plaque in another microscopic field.
2. Loss of the dystrophic structures only, but not the parental axon. Interestingly, in some of these cases, after a variable period of time, new AxDs appeared at the same point of the axon where the previous AxD had been located. This re-formation phenomenon of AxDs always occurred at the edge of the axon (Figs. 3 and 4; Additional file 5; Table 1).

**Fig. 6:**
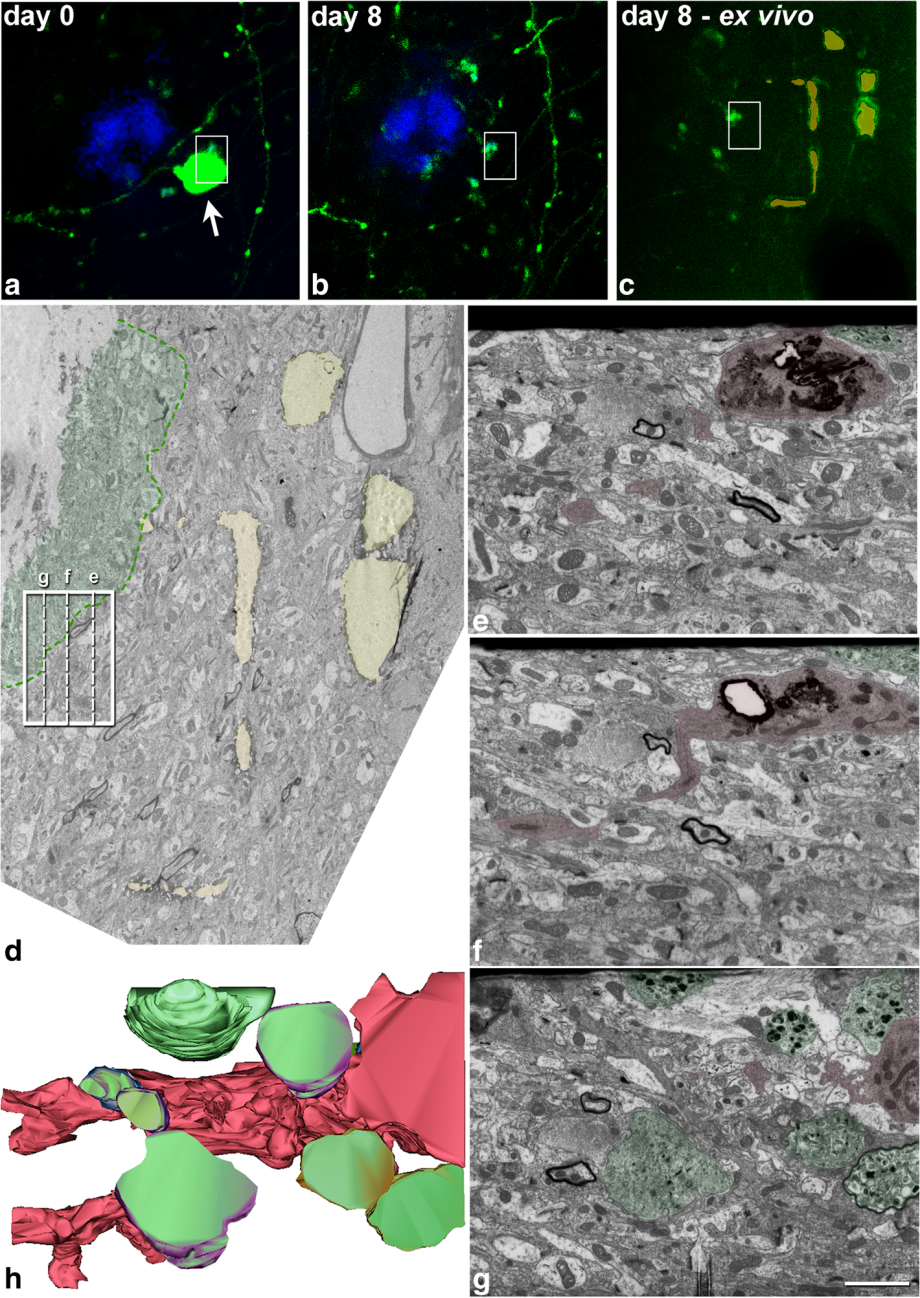
Correlative two-photon in vivo imaging and FIB/SEM microscopy. (**a**, **b**), Optical single plane images taken at two different time points at the same region in layer I of the somatosensory cortex of the dE9 mouse, showing a Methoxy-X04-stained plaque (*blue*) and GFP-expressing processes. There is a loss of an AxD (*arrow*) in the space of 1 week. (**c**), Same region as in **a**, **b** taken ex vivo after the perfusion of the animal on day 8. This image was obtained by the combination of two optical single planes: one taken at the same z-level as in **a**, **b** and the other taken 4 μm above it, showing the NIRB marks (pseudocolored in *yellow* and *orange*) performed to locate the region of interest at the ultrastructural level. (**d**), Electron-micrograph picture from the last ultrathin section taken from the surface of the block that was further analyzed by FIB/SEM microscopy. The same NIRB marks (pseudocolored in *yellow* and *orange*) as in **c** can be seen. The plaque halo is pseudocolored in *green*. The *rectangle* shows the position and the x, z dimensions of the FIB/SEM stack that was obtained. The same *rectangle* is shown in **a**-**c**. The three *dashed lines* inside the rectangle show the perpendicular plane in the approximate region where images **e**-**g** were obtained by FIB/SEM. (**e**-**g**), Examples of FIB/SEM images that were taken in the z = 9.15 μm image stack (305 images of 30 nm thickness). Using Reconstruct software, the AxDs (*green*) and the microglial cell (*red*) that were present in the stack of images were segmented every 2 sections. (**i**), 3D visualization of the segmented elements (microglial cell: *red*, AxDs: *green*). There is an activated microglia cell in the region where the loss of a GFP-expressing AxD was observed. *Scale bar* (in **g**): 18.2 μm in **a**-**c**; 5.1 μm in **d**; 1.8 μm in **e**-**h**

**Fig. 7:**
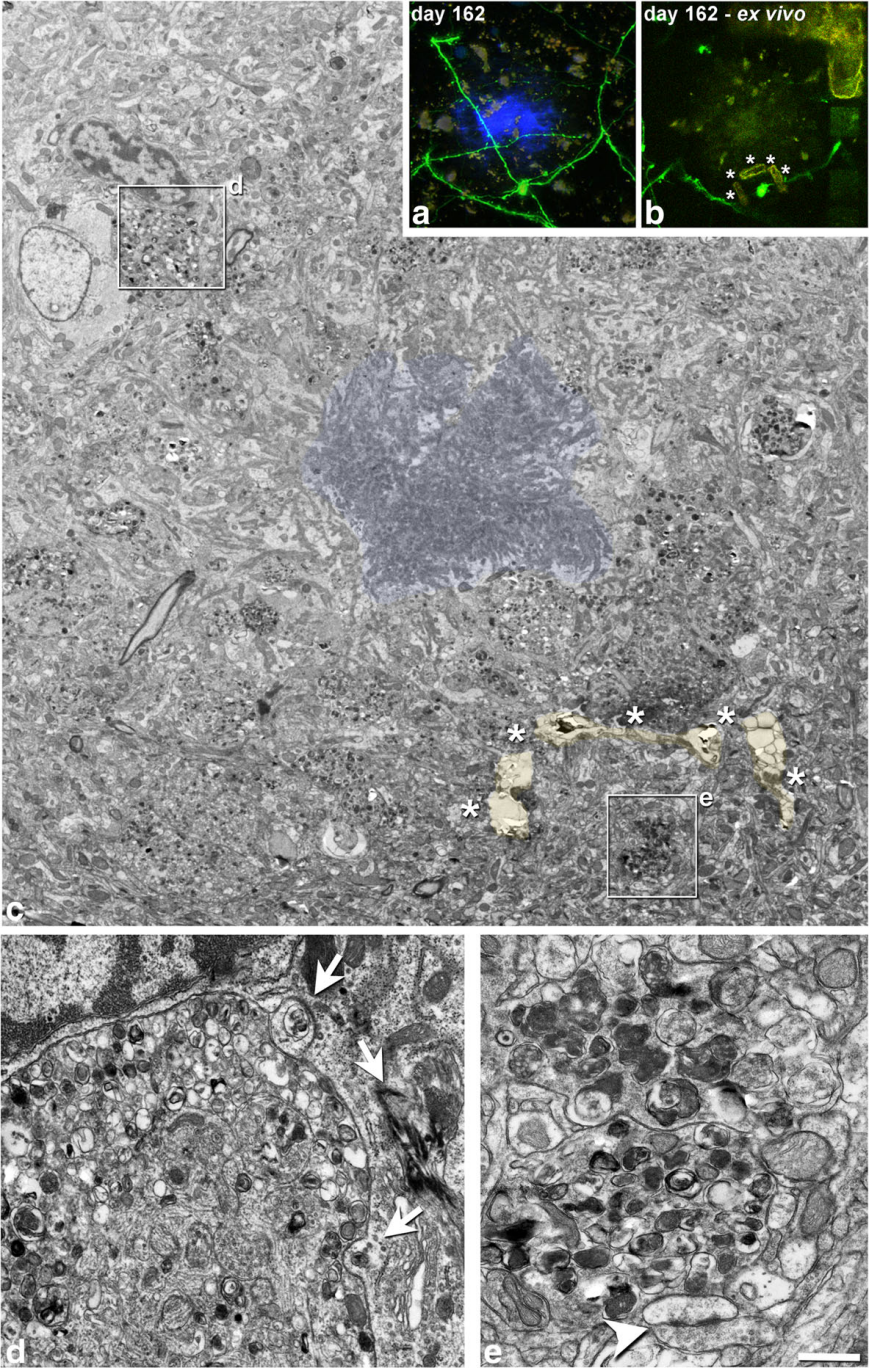
Correlative two-photon microscopy and TEM of an Aβ plaque. (**a**), Maximum projection of images taken from a GFP-expressing AxD near an Aβ plaque stained with Methoxy-X04 (*blue*) on day 162 (the same AxD (dys 7) as in Fig. 1a-c – *red arrow*). (**b**), Ex vivo single plane of the AxD (note that some NIRB marks were made around the region of interest to locate it in subsequent processing steps; marks have been labeled with *asterisks*). (**c**), Low-magnification electron micrograph showing the Aβ plaque (the central core formed by fibrillar Aβ peptide has been pseudocolored in *blue*). The GFP-expressing AxD (dys 7) that was imaged in vivo is surrounded by the laser marks (pseudocolored in *orange* and labeled with *asterisks*). *Rectangles* delimit the regions shown at a higher magnification in panels **d**, **e**. (**d**), Image showing a microglial cell with numerous phagocytic inclusions (*arrows*) in close apposition to an AxD, suggesting that this cell is participating in phagocytosis of the AxD. Microglial cell was identified based on its ultrastructural characteristics. (**e**), Higher magnification of dys 7. Note that autophagic vacuoles take up a large area of the AxD contents, and also that the AxD is almost devoid of any normal-looking organelles. A normal-looking asymmetric synapse can be observed in close apposition to the AxD (*arrowhead*). *Scale bar* (in **e**): 23.1 μm in **a**, **b**; 3 μm in **c**; 0.61 μm in **d**; 0.52 μm in **e**

*Electron microscopy* Using correlative FIB/SEM, we were able to analyze a region of approximately 1200 μm^3^ where an AxD had been observed in vivo to have disappeared (Fig. 6). In this region, at the ultrastructural level, we found an activated microglial cell containing a large amount of electron-dense material, full of phagocytosed fragments of membranes and organelles. This accumulation of electron-dense material corresponded to the auto-fluorescence observed with the two-photon microscope (Additional file 1) and maintained days later at a point where an AxD was lost (Additional file 7). Furthermore, we used correlative two-photon in vivo imaging and TEM of GFP-expressing AxD near to Aβ plaque. We found that in some cases, microglial cells with numerous phagocytic inclusions were in close apposition to AxDs, suggesting that these cells are participating in phagocytosis of AxDs (e.g., see [56]) (Fig. 7d).

#### Type of axon and dystrophic pathology

**Fig. 8:**
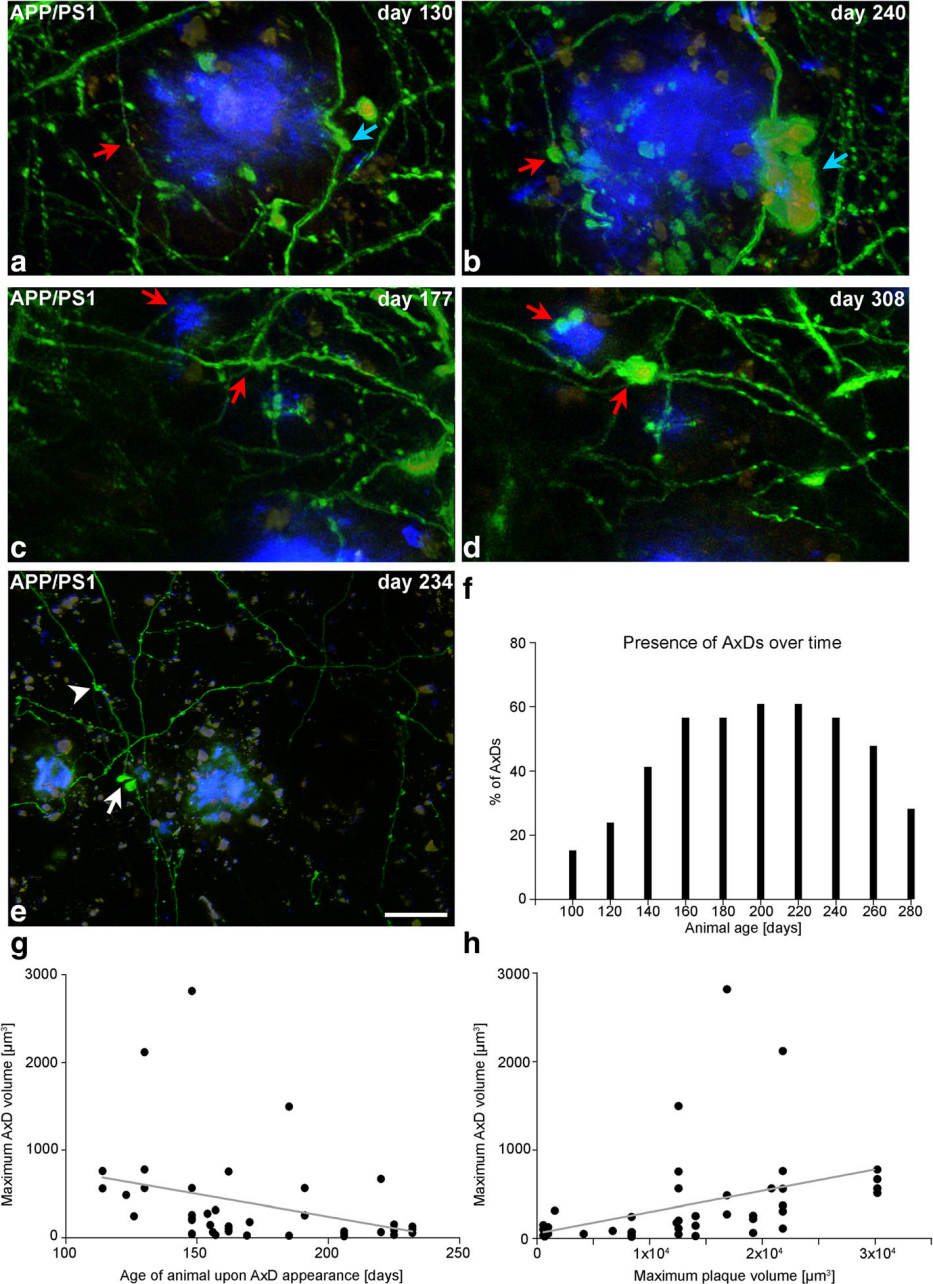
Relationship between Aβ plaques and AxDs formation and development. (**a**, **b**), Maximum projection of images (120 images, z = 0.3 μm) showing the presence of a large AxD (*blue arrow*) and a small AxD (*red arrow*) (GFP, *green*) close to a large plaque stained with Methoxy-X04 (blue) (**b**). Note that the larger AxD starts to develop around day 130 (animal age). (**c**, **d**), Maximum projection of images (120 images, z = 0.3 μm) showing the presence of smaller AxDs (*red arrows*) (GFP, *green*) close to a small Aβ plaque stained with Methoxy-X04 (*blue*) (**d**). These two smaller AxDs appear around day 232 (animal age). (**c**). Panoramic images that show the regions in panels **a** - **d** are in the Additional file 6. (**e**), Maximum projection of images (134 images, z = 0.3 μm) showing GFP-expressing neurites and Aβ plaques stained with Methoxy-X04 (*blue*). There is an axon with two AxDs: one small AxD that is not associated with Aβ plaques (*arrowhead*), and another which is close to an Aβ plaque stained with Methoxy-X04 (*arrow*). The days shown refer to the animal age (the imaging began on day 100 of the animal lifetime). (**f**), Graph showing the percentage of the total number of AxDs (*n* = 47) that are present over time. Days in X correspond to the bin center (bin width = 20 days). (**g**), Correlation (Spearman r: −0.4711, *p* = 0.0022) between the maximum AxD volume and the day of AxD appearance, indicating that larger AxDs develop earlier than smaller AxDs in the animal lifetime. (**h**), Correlation (Spearman r: 0.6173, *p* < 0.0001) between the maximum volume that an AxD reaches and the maximum volume of the Aβ plaque it is associated with. Large AxDs are present only around large Aβ plaques. *Scale bar* (in **e**): 25.1 μm in **a**-**d**; 28.4 μm in **e**

We were also interested in knowing if different types of axons seem to be selectively vulnerable to the amyloid pathology. We distinguished two main types of axons: En Passant Bouton (EPB) axons and Terminal Bouton (TB) axons. EPB axons had a high density of en passant boutons and relatively few spine-like terminal boutons. TB axons were similar to the EPB axons but had a high density of spine-like terminal boutons. Moreover, we could distinguish thin and thick EPB axons. In the dE9 mouse, 80% (8 out of 10) of the AxDs were present in EPB axons (see Fig. 4e–g) and the remaining 20% (2 out of 10) were in TB axons (see Fig. 2). In the APP-PS1 mouse, it was possible to analyze a larger number of AxDs (*n* = 39) (see next section and Additional file 6). In this case, 60% (24 out of 39) of the AxDs were present in EPB axons —Fig. 1d–i; 1 out of these 24 AxDs was present in a thick-EPB axon, see Fig. 8a, b— and the remaining 40% (15 out of 39) were in TB axons (Additional file 4 a–i). In addition, we calculated the number of EPB and TB axons in our images (on day 0 of imaging) in the APP-PS1 mouse model and it was estimated to be 71 and 29%, respectively. Thus, the presence of higher numbers of AxDs in EPB axons could be explained by the higher numbers of EPB axons. When we compared the maximum AxD volume depending on the type of axon, we did not observe significant differences between AxDs in EPB axons (419.6 ± 138.5 μm^3^) and TB axons (374.3 ± 100.4 μm^3^) (Mann–Whitney U: 163.0; *p* = 0.6339). Thus, the size of AxDs was not related to the type of axon in which they were formed (Fig. 3g).

In summary, our data suggest that AxDs were formed in a quarter of GFP-expressing axons, which indicates a selective vulnerability. AxDs, especially those reaching larger sizes, had long lifetimes and appeared as highly plastic structures with large variations in size and shape and axonal sprouting over time. Moreover, they seemed to be formed on different types of axons. Finally, it appeared that microglial cells which are associated with phagocytic activity, may contribute to the disappearance and morphological changes of AxDs. These and other results are summarized in Table 1.

### Relationship between Aβ plaques and AxD formation and development

In order to study early events regarding the formation of AxDs and Aβ plaques, the APP-PS1 mouse was used due to the early Aβ plaque development and high density of Aβ plaques in the neocortex in this mouse compared to the dE9 mouse. Using panoramic and high resolution images (Additional file 6 a-f), it was possible to study 52 AxDs (5 of which were not associated with Aβ plaques) and 33 Aβ plaques (Additional file 6), 15 of which were pre-existing Aβ plaques (Aβ plaques that were already present on the first day of imaging) and 18 were new Aβ plaques (Aβ plaques that appeared during the imaging period).

- The vast majority of AxDs were associated with Aβ plaques and in all these cases the formation of AxDs was observed after the appearance of the Aβ plaques (*n* = 18). However, in a small number of cases we observed an axon with two AxDs (*n* = 5 out of 52); one small AxD that was not associated with Aβ plaques, and another which was close to an Aβ plaque (Fig. 8e).
- There was a correlation (Spearman *r*: 0.6173, *p* < 0.0001) between the maximum size of a given AxD and the maximum size of the Aβ plaque with which it is associated, in such a way that large AxDs were present only around large Aβ plaques, whereas small Aβ plaques were associated only with small AxDs. Nevertheless, both large and small AxDs were present around large Aβ plaques (Fig. 8a-d, h).
- There was a correlation (Spearman *r*: −0.4711, *p* = 0.0022) between the maximum AxD volume and the time of AxD appearance, such that larger AxDs developed earlier than smaller AxDs in the animal lifetime (Fig. 8a-d, g).
- When we studied the distribution of the 47 AxDs associated with Aβ plaques over time, we observed that most of them developed during the imaging period —only 15% of them were present from the first day of imaging (day 100). At the end of the imaging period, around 70% of AxDs had been lost, in most cases due to the disappearance of the whole axon (76%) (Fig. 8f, Additional file 6 a-f).

## Discussion

In our study, we observed that around 22 and 28% of the axons adjacent to Aβ plaques developed dystrophic pathology in the dE9 and APP-PS1, respectively. This is an interesting finding since it indicates that most of the axons near the Aβ plaques are resistant to this pathology. Thus, this raises the question as to why some axons (a minority) seem to be more susceptible than others to the dystrophic pathology.

Using the GFP-M model, it was possible to analyze supragranular layers axons which have been reported [16] to originate mainly from neurons whose soma are located in cortical layers II/III/V (type A3 axon), layer VI (type A2 axon) and thalamus (type A1 axon). These axons are morphologically very similar to thin-EPB, TB and thick-EPB axons, respectively, as defined in the present study. In previous in vivo studies regarding bouton turnover in axons present in layer I of the mouse barrel cortex, it was found that axons from layer VI were highly plastic, followed by axons from layer II/III/V that showed intermediate levels of plasticity and thalamocortical axons in which most of the boutons were persistent [16]. In the present study, we found dystrophic pathology in all these types of axons. However, the majority of axons in contact with Aβ plaques did not develop AxDs. In this regard, it is important to take into account that pyramidal neurons from different layers and even those located in the same layer have different morphological, neurochemical and physiological properties (e.g., [54]). Thus, it is possible that particular types of neurons located in layers II/III/V, layer VI and in the thalamus are more susceptible to developing dystrophic pathology. Indeed, previous studies mainly using a variety of neurochemical markers showed that there are some subpopulations of neurons selectively vulnerable to the AxD development, e.g., those cortico-cortical fibers that express SMI-312 and GAP-43 [37], or neurofilament triplet proteins (NF) [18, 44]. Furthermore, we analyzed only the supragranular layers and it is possible that the susceptibility of the axons in contact with Aβ plaques in layers IV to VI is different to the observations in the present study in the supragranular layers. Thus, further studies are necessary to identify the subpopulation(s) of pyramidal cells that are more susceptible to this pathology.

The relationship between the formation of extracellular Aβ deposits and their associated AxDs remains elusive. For years it has been considered that the formation of AxDs was a consequence of Aβ deposits or microglia activation, but not an active participant in the pathogenesis of the Aβ plaques (see [20]). However, this view is changing and there are increasing data suggesting that beta-secretase 1 (BACE1) elevation and associated Aβ overproduction occurs inside the AxDs [26, 27, 46, 55, 64] (see Additional file 8). Moreover, Aβ is a possible cause of the alterations in axonal transport [50]. If a partial transport defect allows more time for axonal APP processing as suggested [55], then this could generate more Aβ, feeding back into a worsening transport defect and progressive enlargement of both Aβ plaques and AxDs. These facts —together with anomalous mitochondrial function and oxidative stress, autophagy and altered lysosomal processing considered in synthesis with the mechanisms of disrupted axonal transport— suggest that AxDs are an important source of extracellular amyloid deposits (see [20]). The plaque induction of neuritic changes and the contribution of AxDs to Aβ deposition are probably not mutually exclusive and could occur concomitantly even in the same AxD.

Several authors have also contributed to the in vivo study of AxDs, providing information about their formation near Aβ plaques and their probability of recovery after different treatments [13, 15, 23, 53, 59]. However, in these studies, the pathology was followed over relatively short time periods (3 days to 5 weeks maximum) and no detailed morphometric studies were performed. Plasticity and axonal sprouting has been observed around amyloid plaques performing in vitro and ex vivo studies using different IHC markers (e.g., GAP-43) ([38, 40, 47, 49, 65] see [5] for a review). The existence of many growth factors around the Aβ plaques has been studied and sprouting has been proposed as a compensatory mechanism for the synaptic alterations that take place near Aβ plaques [39, 45, 63]. However, this plasticity phenomenon had not been detailed described and quantified in vivo to date. An important advantage of our study was that the AxDs were followed over long periods of time (up to 210 days). A great number of axonal segments were followed and AxDs were studied individually to further analyze the heterogeneous changes between different AxDs and in the same AxD over time (plasticity of AxDs). The individual study of the axons and their dystrophies was possible by means of the GFP-M transgenic model that has a low density of neurons expressing GFP. Moreover, the use of specialized tools allowed the performance of 3D reconstruction and measurement of volumes to carry out a detailed quantitative study. Most AxDs were formed and developed during the imaging period, and numerous AxDs had already disappeared by the end of this period (Additional file 9). We were able to observe that AxDs had long lifetimes (commonly more than 100 days). In the APP-PS1 mouse, amyloid pathology and related dystrophic neuritic changes around the Aβ plaques developed earlier than in the dE9 mouse (Aβ plaque deposition in the neocortex begins around the age of 6 months in the dE9 [30] and 2 months in the APP-PS1 mouse [48]). However, we observed in both models similar findings regarding the high AxD plasticity over time. The most important observations are the following: i) AxDs did not grow steadily —their volume increased and decreased over time, showing dramatic volume differences at distinct time points; ii) AxDs located at the end of the axon could also become fully reversed while the parental axon remained and a new AxD could be generated after a variable period of time; iii) Axonal sprouting was a common event: prominent morphological changes occurred in larger AxDs over time and interestingly the re-growth of long axonal segments from axons that were cut at a dystrophic point was observed in the APP-PS1 mouse. This is interesting since it has been recently observed in vivo that, depending on the cell type, some ablated axons spontaneously re-grow and although they never reconnect to their original targets, axons consistently form new boutons at comparable pre-lesion synaptic densities, implying the existence of intrinsic homeostatic programs, which regulate synaptic numbers on regenerating axons [14]. We cannot rule out the possibility that the absence of this phenomenon in dE9 could suggest that the sample size was not sufficient, or an intrinsic difference of the amyloid pathology between the two mouse lines.

Taking these results together, the existence of this neuronal plasticity (especially around Aβ plaques) increases the likelihood that the synaptic abnormalities associated with the Aβ plaques can be reversed within a certain time window before most AxDs and their axons have disappeared. Thus, this opens up the possibility that early prevention or elimination of the Aβ plaques with appropriate therapeutic strategies might prevent disease progression and promote functional axon regeneration and the recovery of neural circuits.

## Conclusion

AxDs were formed only in a quarter of GFP-expressing axons near Aβ-plaques, which indicates a selective vulnerability. AxDs, especially those reaching larger sizes, had long lifetimes and appeared as highly plastic structures with large variations in size and shape and axonal sprouting over time. We observed that most AxDs were formed and developed during the imaging period, and numerous AxDs had already disappeared by the end of this time. This work is the first in vivo study analyzing quantitatively the high plasticity of the axonal pathology around Aβ plaques. We hypothesized that a therapeutically early prevention of Aβ plaque formation or their growth might halt disease progression and promote functional axon regeneration and the recovery of neural circuits.

## Additional files

Additional file 1: Figure S1. Location of auto-fluorescent spots. (PDF 371 kb)
Additional file 2: Table S1. Table showing the number of imaging positions that was successfully imaged weekly during near 6 months. (PDF 260 kb)
Additional file 3: Figure S2. Correlative light and FIB/SEM microscopy. (PDF 317 kb)
Additional file 4: Figure S3. Size ratio of dystrophic segments. (PDF 393 kb)
Additional file 5: Figure S4. Re-formation of AxDs and sprouting phenomenon (re-growth) in the APP-PS1 mouse. (PDF 341 kb)
Additional file 6: Figure S5. Relationship between Aβ plaques and AxDs formation and development. Quantitative analysis of different types of Aβ plaques. (PDF 463 kb)
Additional file 7: Figure S6. Behavior of auto-fluorescent spots after the elimination of an AxD. (PDF 204 kb)
Additional file 8: Figure S7. Quantitative study of the neurochemical characteristics of the AxDs. (PDF 351 kb)
Additional file 9: Figure S8. Schematic representation of Aβ plaques and AxDs development over time. (PDF 200 kb)

## Abbreviations

3D: Three-dimensional
AD: Alzheimer’s disease
APP-PS1: Amyloid Precursor Protein – Preseniline 1
AxDs: Axonal dystrophies
Aβ: Amyloid β
BP: Bandpass
EPB: En Passant Bouton axons
FIB/SEM: Focused ion beam/scanning electron microscopy
GFP-M: Green fluorescent protein – M
NFTs: Neurofibrillary tangles
NIRB: Near infrared branding
SP: Short pass
TB: Terminal Bouton axons
TEM: Transmission electron microscopy
