# Supplementary material for: High plasticity of axonal pathology in Alzheimer’s disease mouse models
Source: Acta Neuropathol Commun. 2017 Feb 7;5:14. doi: 10.1186/s40478-017-0415-y (PMC5296955; doi:10.1186/s40478-017-0415-y)
Supplement: Additional file 1: Figure S1. — Location of auto-fluorescent spots. (PDF 371 kb) [file 40478_2017_415_MOESM1_ESM.pdf]

SUPPLEMENTARY FIGURE 1

High plasticity of axonal pathology in Alzheimer's disease mouse models

Lidia Blazquez-Llorca[a,+,*], Susana Valero-Freitag[a,+], Eva Ferreira Rodrigues[a], Ángel Merchán-Pérez[b,c], J. Rodrigo Rodríguez[b,d], Mario M. Dorostkar[a], Javier DeFelipe[b,d,e] and Jochen Herms[a,f*]

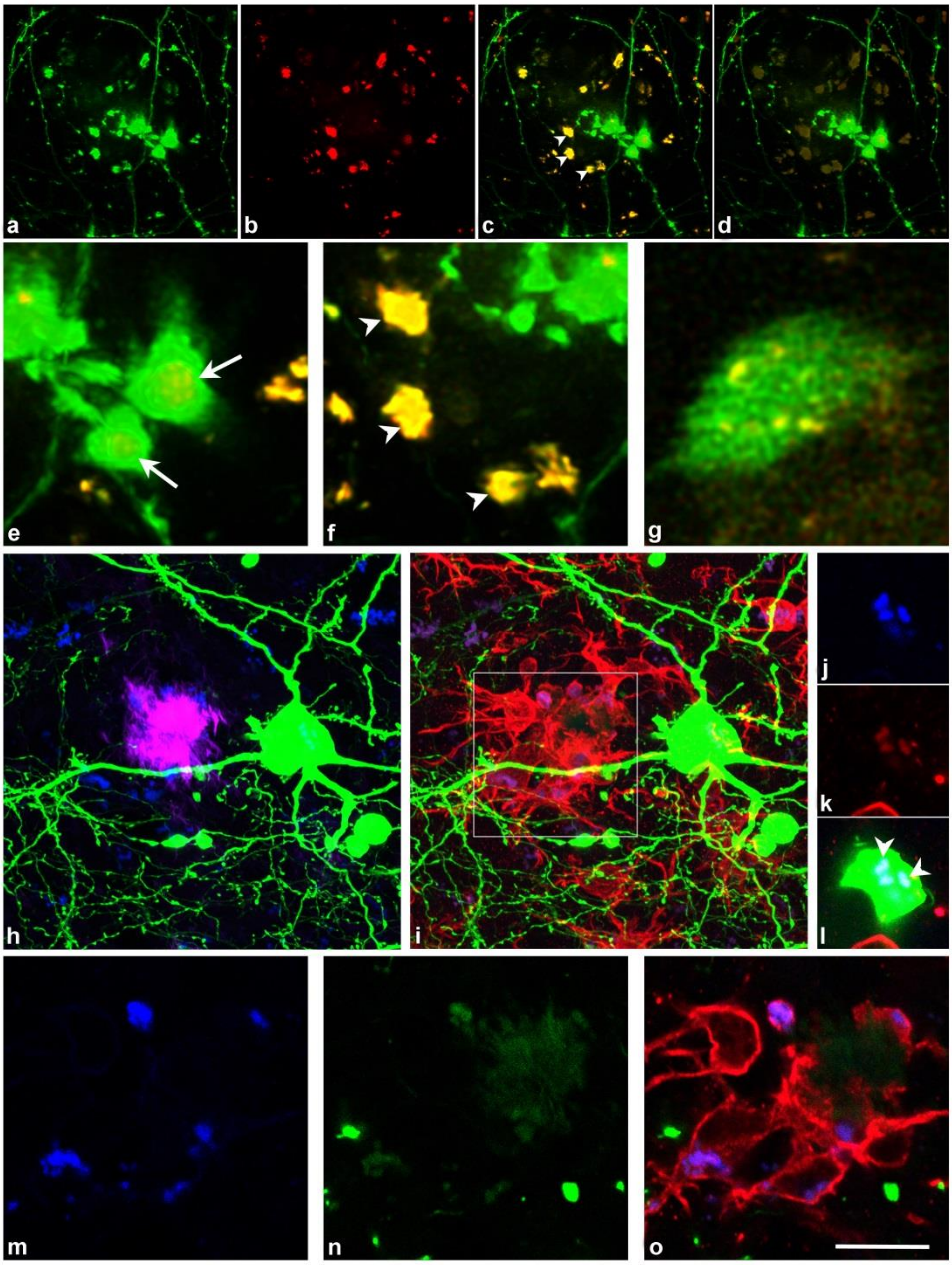

Supplementary Fig. 1 Location of auto-fluorescent spots. (**a–d**), Two-photon *in vivo* images of the same GFP-expressing AxD (dys 1) around an Aβ plaque stained with Methoxy-X04 (blue) in the somatosensory cortex of the dE9 mouse (the same AxD is shown in Fig. 2). Two-photon excitation of GFP-expressing neuronal structures was performed at 880 nm and the signal was detected using a bandpass (BP) 500–550 nm filter (**a**). To exclude false positive fluorescent spots from the analysis, we also recorded emissions at 590–650 nm (**b**). By combining both images, the auto-fluorescent spots can be visualized as yellow structures (arrowheads, the same as in **f**) (**c**). When necessary, Photoshop was used to facilitate the visualization of GFP-expressing structures by manually darkening those auto-fluorescent structures that were alone in the neuropil and not inside any GFP-expressing structure (**d**). (**e–g**), Higher magnification of two-photon *in vivo* images showing that the auto-fluorescent spots can be inside GFP-expressing AxDs (arrow, **E**), in the neuropil (arrowheads, **f**) and inside neuronal cell bodies (lipofuscin, **g**). (**h–o**), Immunohistochemistry for Iba-1 (red) was performed to better define the presence of the auto-fluorescence. (**h**), Maximum projection of images (44 images, z = 0.4 μm) showing a GFP-expressing pyramidal neuron and numerous neuronal processes (green) near an Aβ plaque stained with Methoxy-X04 (magenta). The far-red fluorescence was also recorded to facilitate the visualization of auto-fluorescent spots (blue). (**I**), Same stack of images and field of view as in **h** adding the fluorescence recorded for Alexa 594 (immunofluorescence of Iba-1, red) and excluding the fluorescence recorded for Methoxy-X04 (Aβ plaque, magenta). (**j–l**), Single optical section showing a higher magnification of the neuronal body of the pyramidal neuron in (**h, i**). Auto-fluorescent spots (recorded with far-red fluorescence (**j**), Alexa 594 (**k**) and GFP channel (**l**)) are inside the neuronal cell body (lipofuscin (white), arrowheads) (**l**). (**m–o**), Single optical section showing a higher magnification of the region in **i** that was delimited by a square. Auto-fluorescent spots (arrowheads, recorded with far-red (**m**), GFP (**n**) and Alexa 594 channel (**o**)) are enclosed within microglial processes (red) (**o**). Scale bar (in **o**): 25.5 μm in **a–d**; 5.2 μm in **e–g**; 12.5 μm in **h, i**; 6 μm in **j–l**; 6.3 μm in **m–o**
