# Supplementary material for: High plasticity of axonal pathology in Alzheimer’s disease mouse models
Source: Acta Neuropathol Commun. 2017 Feb 7;5:14. doi: 10.1186/s40478-017-0415-y (PMC5296955; doi:10.1186/s40478-017-0415-y)
Supplement: Additional file 2: Table S1. — Table showing the number of imaging positions that was successfully imaged weekly during near 6 months. (PDF 260 kb) [file 40478_2017_415_MOESM2_ESM.pdf]

SUPPLEMENTARY TABLE 1

High plasticity of axonal pathology in Alzheimer's disease mouse models

Lidia Blazquez-Llorca[a,+,*], Susana Valero-Freitag[a,+], Eva Ferreira Rodrigues[a], Ángel Merchán-Pérez[b,c], J. Rodrigo Rodríguez[b,d], Mario M. Dorostkar[a], Javier DeFelipe[b,d,e] and Jochen Herms[a,f*]

Images properties	Aim	Mouse model	Number of imaging positions	Number of time points / Total number of images	Number of dystrophic axons	Total number of axons	Number of Aβ plaques
High magnification images (logical size 512 × 512 pixels; physical size x, y, z: 84.9 x 84.9 x 40□60 μm; z-step = 1 μm)	The 3D reconstruction of AxDs over time	dE9 (number of animals, n = 6)	#1	19 / 19	1	6	1
			#2	19 / 19	4	11	1
			#3	19 / 19	1	5	1
			#4	19 / 19	1	8	1
			#5	18 / 18	2	10	1
			#6	18 / 18	1	6	1
			**TOTAL**	**18 - 19 / 112**	**10**	**46**	**6**
		APP-PS1 (number of animals, n = 7)	#1	17 / 17	3	9	1
			#2	23 / 23	5	14	1
			#3	23 / 23	3	9	1
			#4	23 / 23	3	11	1
			#5	19 / 19	1	8	1
			#6	19 / 19	1	7	1
			**TOTAL**	**17 - 23 / 124**	**16**	**58**	**6**
Panoramic high-resolution images (logical size 1400 × 1400 pixels; physical size x, y, z: 202.3 x 202.3 x 39.9□50.1 μm; z-step = 0.3 μm)*	The spatiotemporal relationship between Aβ plaques and AxDs	APP-PS1 (number of animals, n = 7)	#1	23 / 23	14	-	6
			#2	23 / 23	6	-	4
			#3	23 / 23	3	-	2
			#4	23 / 23	9	-	7
			#5	23 / 23	11	-	6
			#6	21 / 21	4	-	4
			#7	17 / 17	5	-	4
			**TOTAL**	**17 - 23 / 153**	**52**	**-**	**33**

Supplementary Table 1 Table showing the number of imaging positions that was successfully weekly imaged during near 6 months. For each imaging position is also shown: the number of images acquired over time (time points), and the number of dystrophic axons, axons and Aβ plaques present in each imaging position.
Two types of images were acquired to carry out the aims of the work.
*Note that data from panoramic high-resolution images (lifetime of AxDs and type of axon where AxDs are formed) were also used in Aim "The 3D reconstruction of AxDs over time".
