# Supplementary material for: High plasticity of axonal pathology in Alzheimer’s disease mouse models
Source: Acta Neuropathol Commun. 2017 Feb 7;5:14. doi: 10.1186/s40478-017-0415-y (PMC5296955; doi:10.1186/s40478-017-0415-y)
Supplement: Additional file 3: Figure S2. — Correlative light and FIB/SEM microscopy. (PDF 317 kb) [file 40478_2017_415_MOESM3_ESM.pdf]

SUPPLEMENTARY FIGURE 2

High plasticity of axonal pathology in Alzheimer's disease mouse models

Lidia Blazquez-Llorca[a,+,*], Susana Valero-Freitag[a,+], Eva Ferreira Rodrigues[a], Ángel Merchán-Pérez[b,c], J. Rodrigo Rodríguez[b,d], Mario M. Dorostkar[a], Javier DeFelipe[b,d,e] and Jochen Herms[a,f*]

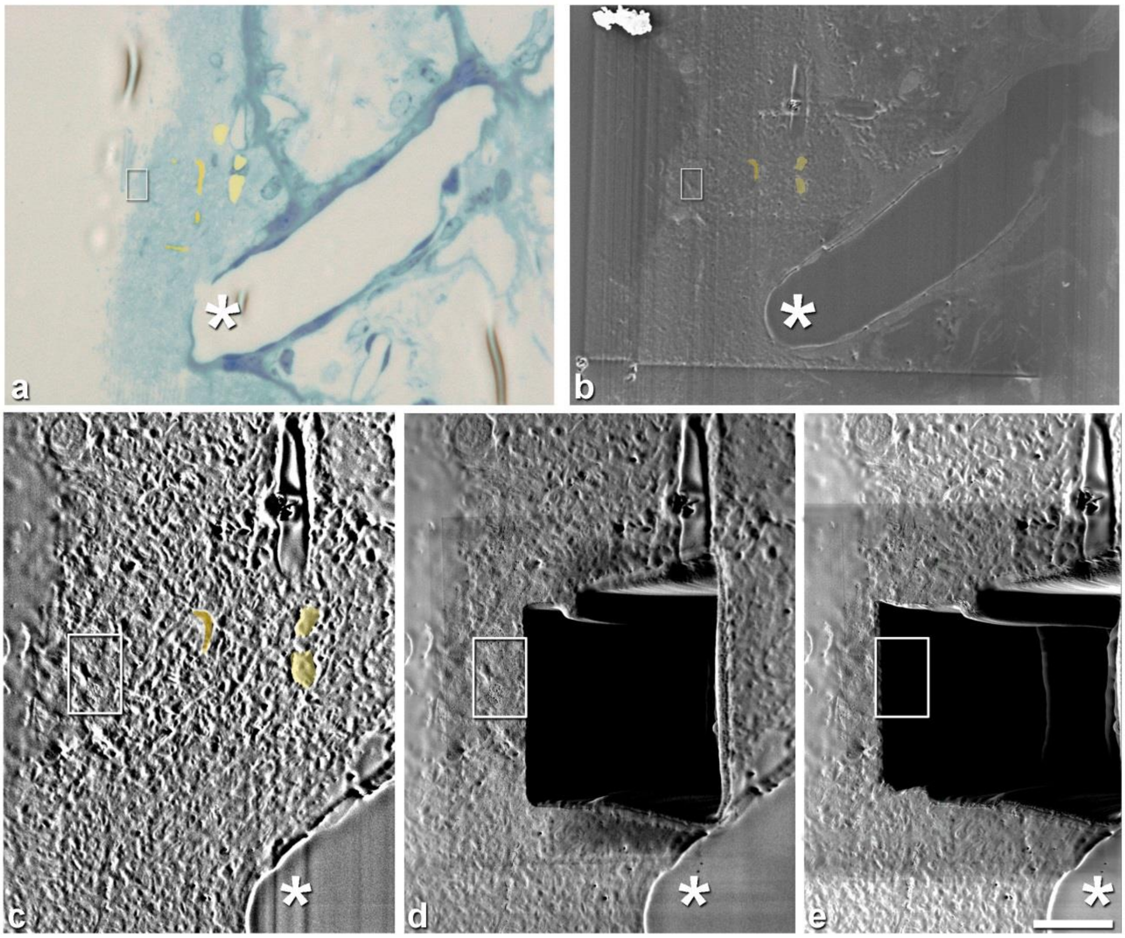

Supplementary Fig. 2 Correlative light and FIB/SEM microscopy. (**a**)**,** Photograph of the final Toluidine blue stained semithin section that was taken from the surface of the block containing the region of interest shown in Fig. 6 and that was further analyzed using FIB/SEM microscopy. NIRB marks are visible (pseudocolored in yellow and orange; see Fig, 6 c). (**b**), Same field of view as in **a** showing the correlative laser marks on the surface of the block used for FIB/SEM (pseudocolored in yellow and orange). (**c–d**), Higher SEM image magnification of the region of interest (rectangle), before (**c**) and after (**d**) milling of the trench needed to obtain back-scattered electron images. **d** shows the beginning of acquisition of the stack of electron microscopy images and **e** shows the trench after the region of interest has been fully reconstructed. The rectangle in all images shows the position and the x, z dimensions of the FIB/SEM stack that was obtained. The asterisk points out the same blood vessel. Scale bar (in **e**): 44.5 μm in **a, b**; 15 μm in **c–e**
