# Supplementary material for: High plasticity of axonal pathology in Alzheimer’s disease mouse models
Source: Acta Neuropathol Commun. 2017 Feb 7;5:14. doi: 10.1186/s40478-017-0415-y (PMC5296955; doi:10.1186/s40478-017-0415-y)
Supplement: Additional file 4: Figure S3. — Size ratio of dystrophic segments. (PDF 393 kb) [file 40478_2017_415_MOESM4_ESM.pdf]

SUPPLEMENTARY FIGURE 3

High plasticity of axonal pathology in Alzheimer's disease mouse models

Lidia Blazquez-Llorca[a,+,*], Susana Valero-Freitag[a,+], Eva Ferreira Rodrigues[a], Ángel Merchán-Pérez[b,c], J. Rodrigo Rodríguez[b,d], Mario M. Dorostkar[a], Javier DeFelipe[b,d,e] and Jochen Herms[a,f*]

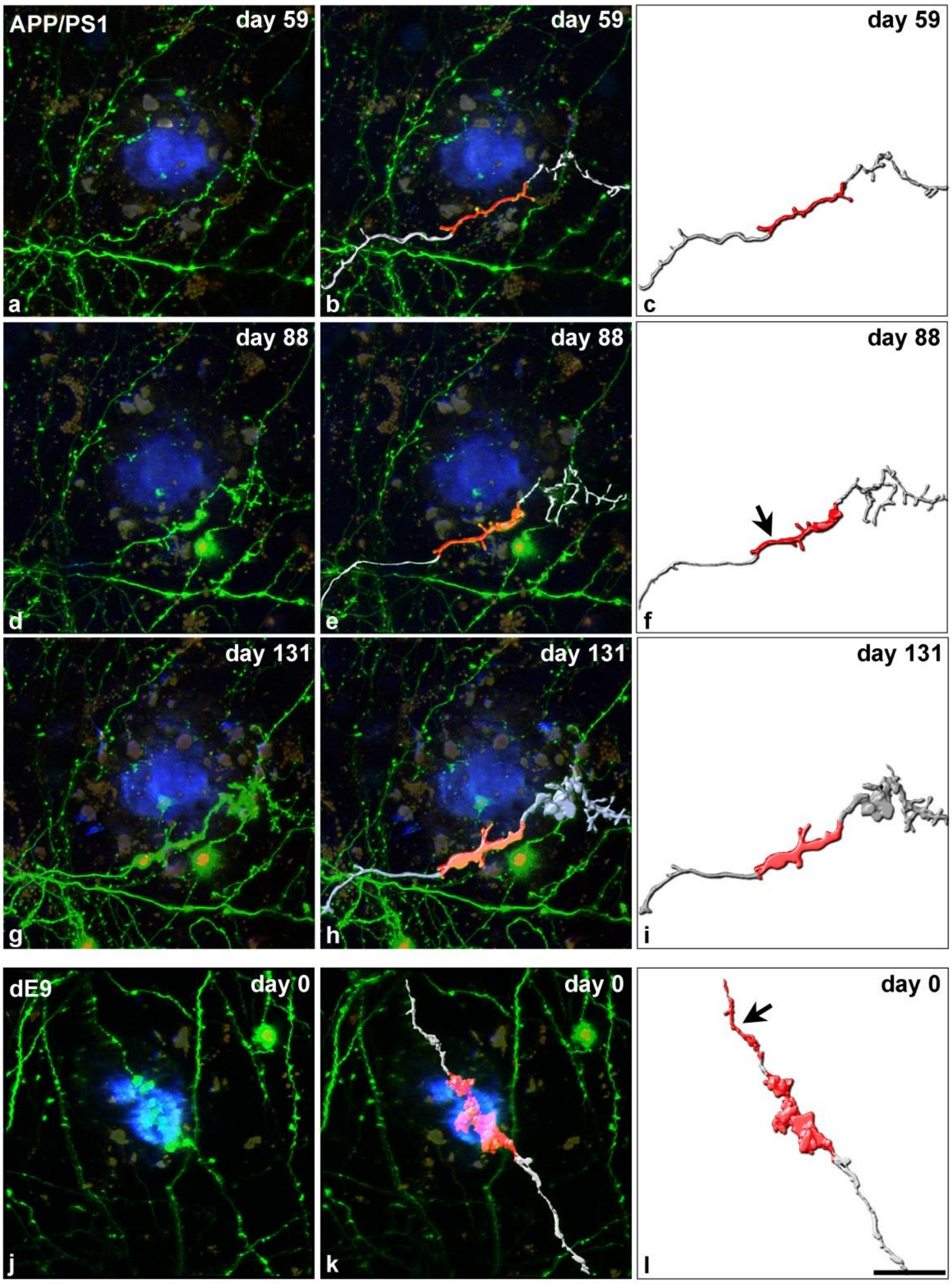

Supplementary Fig. 3 Size ratio of dystrophic segments. (**a–i**)**,** Two-photon *in vivo* images of a GFP-expressing AxD (dys 7) near an Aβ plaque stained with Methoxy-X04 (blue) in the somatosensory cortex of the APP-PS1 mouse at three different time points (**a**, **d**, **g**). The axon was reconstructed using Imaris software (**b**, **e**, **h**, respectively). The reconstruction can be observed in isolation in **c, f, i**, respectively. The dystrophic segment is shown in red. To obtain the size ratio: the "normal axon volume" was determined by taking, if possible, the average volume of 3 axonal segments at three different time points, prior to the AxD formation. Segments were of the same length and in the same position as the maximum AxD segment that will later appear (**c**). In **i**, the maximum AxD segment can be observed. Note that in **f** the region pointed out by the arrow is still non-dystrophic. (**j–l**), Two-photon *in vivo* images of a GFP-expressing AxD (dys 1) around an Aβ plaque stained with Methoxy-X04 (blue) in the somatosensory cortex of the dE9 mouse (same as in Fig. 2). The axon was reconstructed using Imaris software (**k**). The reconstruction can be observed in isolation in **l**. The dystrophic segment is shown in red, as is the "normal-looking axon segment" (arrow). In those cases where the AxD was present from the first day of imaging, the segments taken as "normal" were those that were from the same normal-looking axon, with the same length as the maximum AxD segment and which were situated outside the Aβ plaque. Scale bar (in **l**): 23.1 μm in **a–l**
