# Supplementary material for: High plasticity of axonal pathology in Alzheimer’s disease mouse models
Source: Acta Neuropathol Commun. 2017 Feb 7;5:14. doi: 10.1186/s40478-017-0415-y (PMC5296955; doi:10.1186/s40478-017-0415-y)
Supplement: Additional file 5: Figure S4. — Re-formation of AxDs and sprouting phenomenon (re-growth) in the APP-PS1 mouse. (PDF 341 kb) [file 40478_2017_415_MOESM5_ESM.pdf]

SUPPLEMENTARY FIGURE 4

High plasticity of axonal pathology in Alzheimer's disease mouse models

Lidia Blazquez-Llorca[a,+,*], Susana Valero-Freitag[a,+], Eva Ferreira Rodrigues[a], Ángel Merchán-Pérez[b,c], J. Rodrigo Rodríguez[b,d], Mario M. Dorostkar[a], Javier DeFelipe[b,d,e] and Jochen Herms[a,f *]

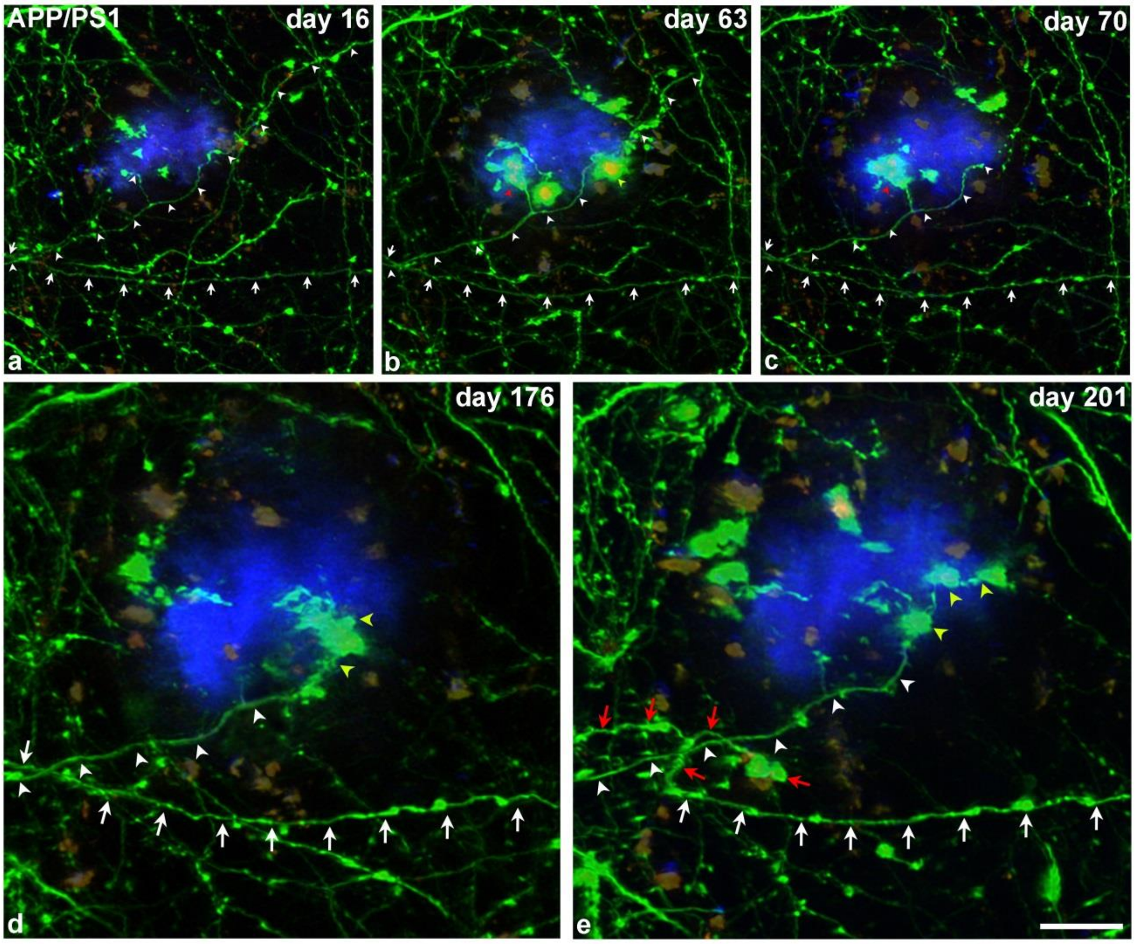

Supplementary Fig. 4 Re-formation of AxDs and sprouting phenomenon (re-growth) in the APP-PS1 mouse. (**a**–**e**), Maximum projection of a stack of images (40 images; z-step: 1 μm) taken with the two-photon microscope in the supragranular layers of the somatosensory cortex of the APP-PS1 mouse at five different time points showing some neurites expressing GFP around an Aβ plaque stained with Methoxy-X04 (blue). There are two axons of interest, one is marked with arrowheads (axon 1) and the other with arrows (axon 2). Axon 1: At the beginning, axon 1 looks normal (**a**). Days later, the middle part of the axon becomes dystrophic (yellow arrowhead – dys 2a), as does a short branch (red arrowhead – dys 1). Moreover, a distal portion of axon 1 disappears and so the axon as a whole is shortened (**b**). One week later, the AxD in the middle part has disappeared and axon 1 is cut at this point (**c**). Days later, the AxD at the short branch (dys 1) has disappeared and another AxD (dys 2b) appears at the edge of axon 1 where dys 2a was previously present (**d**). The morphology of this AxD changes over time (**e**). Axon 2: At the beginning, axon 2 looks normal (**a**–**b**). Days later, this axon gets thicker (**c**–**d**) and, at some point, the axon is cut and then re-grows through the Aβ plaque. The new re-growth segment (32 μm) becomes dystrophic (**e,** red arrows). Scale bar (in **e**): 19.5 μm in **a**–**c;** 10.3 μm in **d**–**e**
