# Supplementary material for: High plasticity of axonal pathology in Alzheimer’s disease mouse models
Source: Acta Neuropathol Commun. 2017 Feb 7;5:14. doi: 10.1186/s40478-017-0415-y (PMC5296955; doi:10.1186/s40478-017-0415-y)
Supplement: Additional file 6: Figure S5. — Relationship between Aβ plaques and AxDs formation and development. Quantitative analysis of different types of Aβ plaques. (PDF 463 kb) [file 40478_2017_415_MOESM6_ESM.pdf]

SUPPLEMENTARY FIGURE 5

High plasticity of axonal pathology in Alzheimer's disease mouse models

Lidia Blazquez-Llorca[a,+,*], Susana Valero-Freitag[a,+], Eva Ferreira Rodrigues[a], Ángel Merchán-Pérez[b,c], J. Rodrigo Rodríguez[b,d], Mario M. Dorostkar[a], Javier DeFelipe[b,d,e] and Jochen Herms[a,f*]

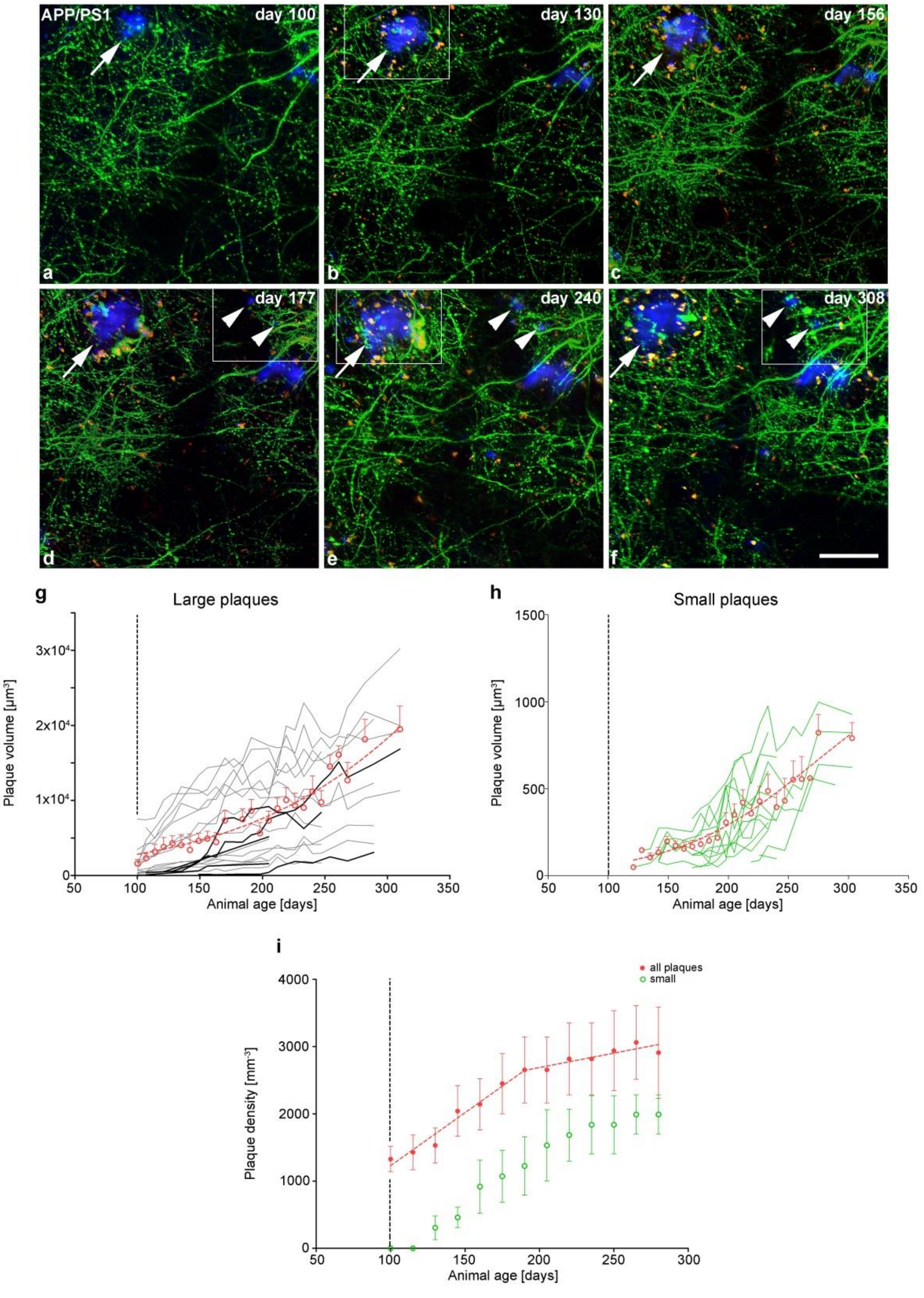

Supplementary Fig. 5 Relationship between Aβ plaques and AxDs formation and development. Quantitative analysis of different types of Aβ plaques. (**a**-**f**), Maximum projection of images (136 images, z = 0.3 µm) showing GFP-expressing neurites and the formation and growth of different types of Aβ plaques over time in the supragranular layers of the somatosensory cortex of the APP-PS1 mouse (two-photon microscopy). An Aβ plaque that reaches a large volume is pointed out by an arrow. Arrowheads point out small plaques. Rectangles in **b**, **e** surround the region shown in Fig. 8 a, b, respectively. Rectangles in **d**, **f** surround the region shown in Fig. 8 c, d, respectively. Note that most AxDs were formed and developed during the imaging period, and numerous AxDs had already disappeared by the end of this time (see Aβ plaque pointed out by an arrow). (**g, h**), Graphs showing the volume of large Aβ plaques (both pre-existing plaques, gray lines; and plaques that appeared during the imaging period, black lines) (**g**) and small Aβ plaques (**h**) in the animal lifetime. Open red circles show the mean ± SEM volume over time for large (**g**) and small (**h**) Aβ plaques. Dashed red line shows the fitted sigmoid curve for large (R^2 0.36) and small Aβ plaques (R^2 0.45): the inflection point of the fitted curve dividing the cubic and asymptotic parts of the function is expected at day 408 in **g** and at day 271 in **h**. (**i**), Graph showing the density of Aβ plaques in the animal age (mean ± SEM) (red circles). Dashed red lines correspond to the two fitted regression lines (R^2 0.28); both lines intercept at day 190. The slope is 15.75 ± 6.34 µm³/day for the first line and 4.28 ± 5.45 µm³/day for the second line. The density of small Aβ plaques against animal age (mean ± SEM) (green open circles) has been also represented. Scale bar (in **f**): 42.7 µm in **a**–**f**
