# Supplementary material for: High plasticity of axonal pathology in Alzheimer’s disease mouse models
Source: Acta Neuropathol Commun. 2017 Feb 7;5:14. doi: 10.1186/s40478-017-0415-y (PMC5296955; doi:10.1186/s40478-017-0415-y)
Supplement: Additional file 7: Figure S6. — Behavior of auto-fluorescent spots after the elimination of an AxD. (PDF 204 kb) [file 40478_2017_415_MOESM7_ESM.pdf]

**SUPPLEMENTARY FIGURE 6**

# High plasticity of axonal pathology in Alzheimer's disease mouse models

Lidia Blazquez-Llorca[a,+,*], Susana Valero-Freitag[a,+], Eva Ferreira Rodrigues[a], Ángel Merchán-Pérez[b,c], J. Rodrigo Rodríguez[b,d], Mario M. Dorostkar[a], Javier DeFelipe[b,d,e] and Jochen Herms[a,f*]

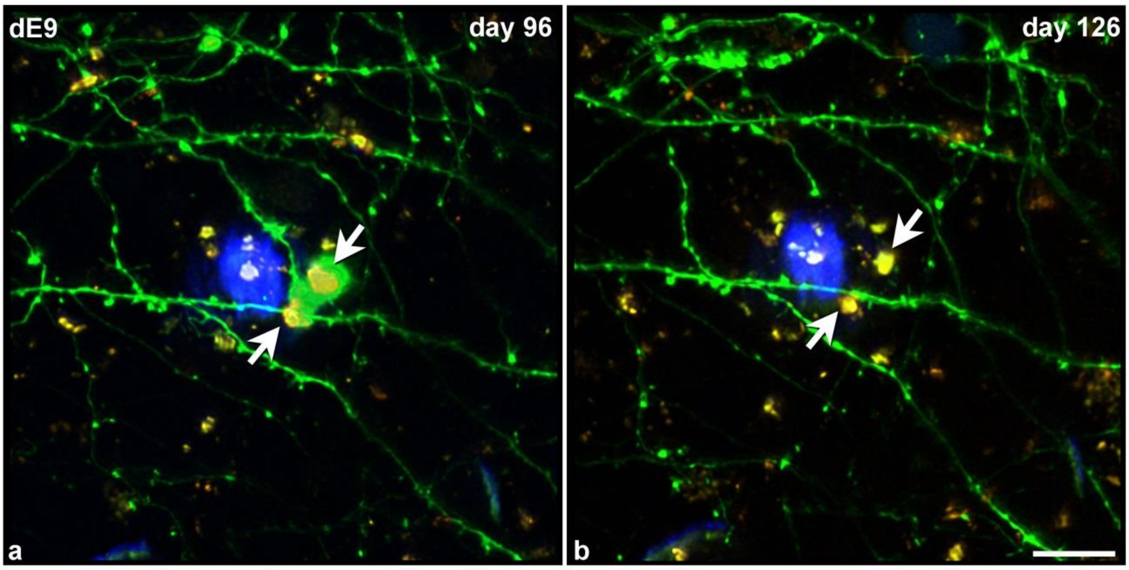


**Supplementary Fig. 6 Behavior of auto-fluorescent spots after the elimination of an AxD.** (**a, b**), Maximum projection of a stack of images (40 images**;** z-step: 1 μm) taken with the two-photon microscope in the supragranular layers of the somatosensory cortex of the dE9 mouse at two different time points, showing some neurites expressing GFP around an Aβ plaque stained with Methoxy-X04 (blue). This AxD is the same one showed in Fig. 4. It can be observed that the auto-fluorescence (yellow spots) inside the GFP-expressing AxD (in **a**) remains at the same location after the loss of the AxD (in **b**). The arrows point out the region of interest. Scale bar (in **b**): 12.4 μm in **a, b**
