# Supplementary material for: High plasticity of axonal pathology in Alzheimer’s disease mouse models
Source: Acta Neuropathol Commun. 2017 Feb 7;5:14. doi: 10.1186/s40478-017-0415-y (PMC5296955; doi:10.1186/s40478-017-0415-y)
Supplement: Additional file 8: Figure S7. — Quantitative study of the neurochemical characteristics of the AxDs. (PDF 351 kb) [file 40478_2017_415_MOESM8_ESM.pdf]

**SUPPLEMENTARY FIGURE 7**

# High plasticity of axonal pathology in Alzheimer's disease mouse models

Lidia Blazquez-Llorca[a,+,*], Susana Valero-Freitag[a,+], Eva Ferreira Rodrigues[a], Ángel Merchán-Pérez[b,c], J. Rodrigo Rodríguez[b,d], Mario M. Dorostkar[a], Javier DeFelipe[b,d,e] and Jochen Herms[a,f *]

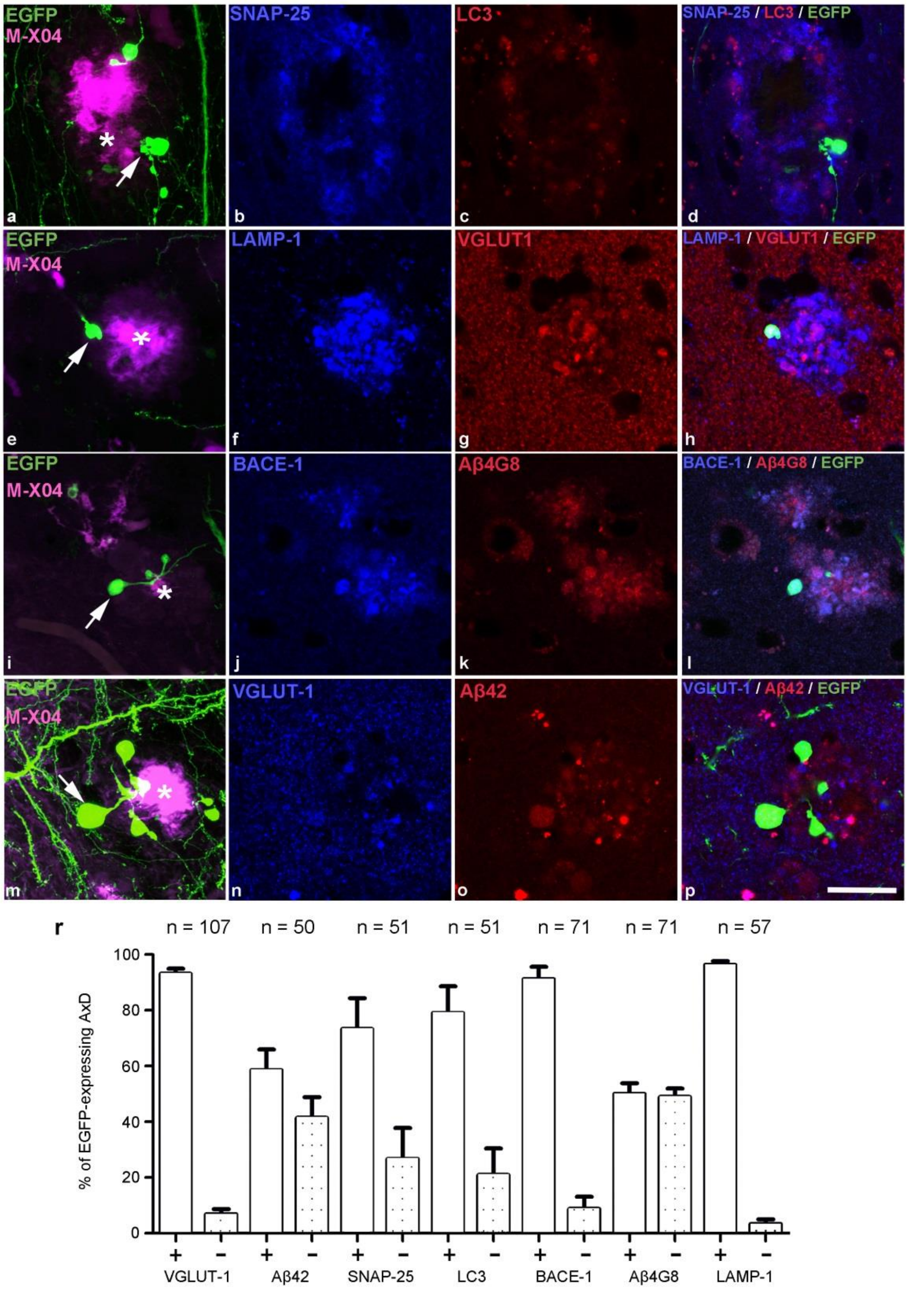


**Supplementary Fig. 7 Quantitative study of the neurochemical characteristics of the AxDs.** Confocal images of the supragranular layers of the somatosensory cortex in coronal sections of the dE9 mouse brain (~ 12 months) in which immunohistochemistry was performed with different antibodies. (**a, e, i, m**), Maximum projection (z = 0.4 μm) to display the AxDs present in axons of pyramidal neurons expressing GFP (white arrows) in close proximity to an Aβ plaque stained with Methoxy-X04 (magenta, white asterisks). (**b, c**), Single plane of the stack taken in the region in **a** showing immunoreactivity for SNAP-25 (blue) and LC3 (red), respectively. (**f, g**), Single plane of the stack taken in the region in **e** showing immunoreactivity for LAMP1 (blue) and VGLUT 1 (red), respectively. (**j, k**), Single plane of the stack taken in the region in **I** showing immunoreactivity for BACE 1 (blue) and Aβ4G8 (red), respectively. (**n, o**), Single plane of the stack taken in the region in **m** representing immunoreactivity for VGLUT-1 (blue) and Aβ42 (red), respectively. (**d, h, l, p**), Same optical plane in **b**–**c**, **f**–**g**, **j**–**k** and **n**–**o**, respectively, in which the image showing the expression of GFP in this plane has been digitally added. (**r**), Quantification of the expression of VGLUT-1, Aβ42, SNAP25, LC3, BACE1, Aβ4G8 and LAMP1 in AxDs expressing GFP in the dE9 mouse. Scale bar (in **p**): 21.7 μm in **a**–**p**
