# Supplementary material for: High plasticity of axonal pathology in Alzheimer’s disease mouse models
Source: Acta Neuropathol Commun. 2017 Feb 7;5:14. doi: 10.1186/s40478-017-0415-y (PMC5296955; doi:10.1186/s40478-017-0415-y)
Supplement: Additional file 9: Figure S8. — Schematic representation of Aβ plaques and AxDs development over time. (PDF 200 kb) [file 40478_2017_415_MOESM9_ESM.pdf]

SUPPLEMENTARY FIGURE 8

High plasticity of axonal pathology in Alzheimer's disease mouse models

Lidia Blazquez-Llorca[a,+,*], Susana Valero-Freitag[a,+], Eva Ferreira Rodrigues[a], Ángel Merchán-Pérez[b,c], J. Rodrigo Rodríguez[b,d], Mario M. Dorostkar[a], Javier DeFelipe[b,d,e] and Jochen Herms[a,f*]

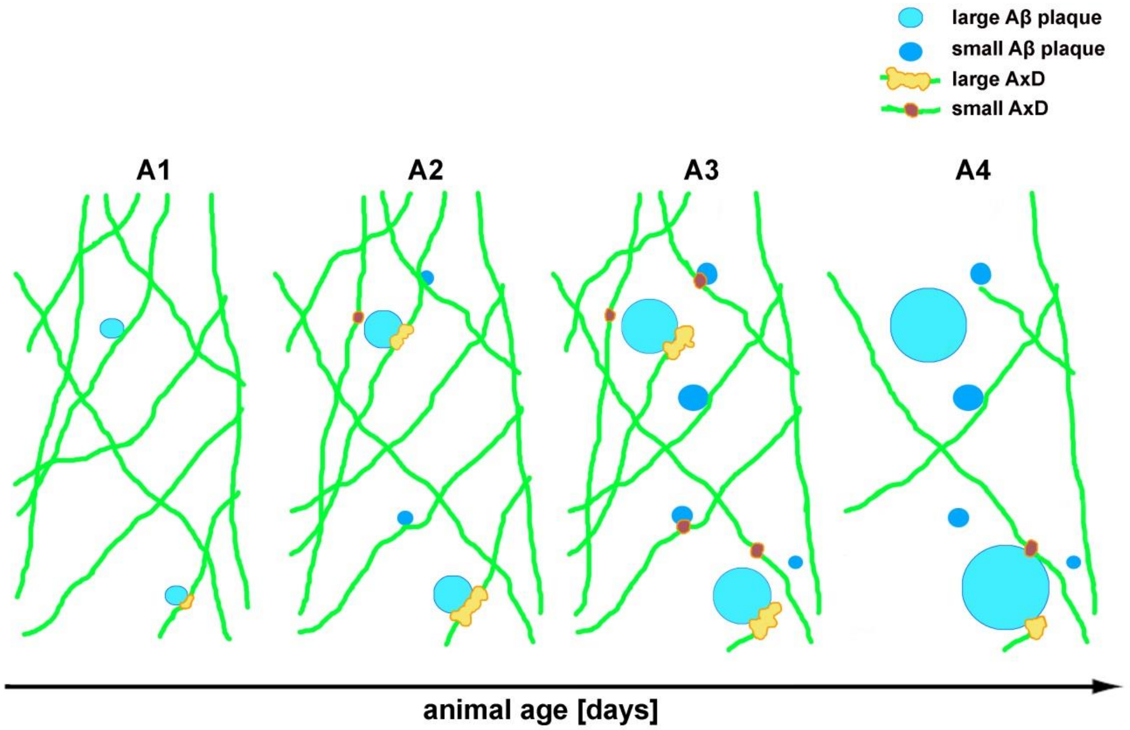

Supplementary Fig. 8 Schematic representation of Aβ **plaques and AxDs development over time.** Initially, Aβ plaques develop (A1). Larger AxDs appear associated with Aβ plaques that will become large (A1, A2). Smaller Aβ plaques are formed next to pre-existing large Aβ plaques. Small AxDs develop around both small and large Aβ plaques. Large Aβ plaque volume increases rapidly (A3). Finally, numerous axons and AxDs disappear. Large Aβ plaques continue increasing their volume whereas plaque density is stabilized (A4). Neurites are represented in green
